# Aminotransferase SsAro8 Regulates Tryptophan Metabolism Essential for Filamentous Growth of Sugarcane Smut Fungus *Sporisorium scitamineum*

**DOI:** 10.1128/spectrum.00570-22

**Published:** 2022-07-06

**Authors:** Guobing Cui, Chengwei Huang, Xinping Bi, Yixu Wang, Kai Yin, Luyuan Zhu, Zide Jiang, Baoshan Chen, Yi Zhen Deng

**Affiliations:** a State Key Laboratory for Conservation and Utilization of Subtropical Agro-Bioresources/Guangdong Province Key Laboratory of Microbial Signals and Disease Control/Integrative Microbiology Research Centre, South China Agricultural University, Guangzhou, China; b Guangdong Laboratory for Lingnan Modern Agriculture, Guangzhou, China; c College of Life Science and Technology/State Key Laboratory for Conservation and Utilization of Subtropical Agro-Bioresources, Guangxi University, Nanning, China; Septomics Research Center, Friedrich Schiller University and Leibniz Institute for Natural Product Research and Infection Biology—Hans Knöll Institute

**Keywords:** Aro8, *Sporisorium scitamineum*, aromatic alcohols, filamentous growth, fungus, pathogenicity, sugarcane smut, tryptophol

## Abstract

Sugarcane smut caused by the basidiomycetous fungus Sporisorium scitamineum leads to severe economic losses globally. Sexual mating/filamentation of S. scitamineum is critical for its pathogenicity, as only the dikaryotic hyphae formed after sexual mating are capable of invading the host cane. Our comparative transcriptome analysis showed that the mitogen-activated protein kinase (MAPK) pathway and the AGC kinase Agc1 (orthologous to yeast Rim15), both governing *S. scitamineum* mating/filamentation, were induced by elevated tryptophol level, supporting a positive regulation of *S. scitamineum* mating/filamentation by tryptophol. However, the biosynthesis pathway of tryptophol remains unknown in *S. scitamineum*. Here, we identified an aminotransferase orthologous to the established tryptophan aminotransferase Tam1/Aro8, catalyzing the first step of tryptophan-dependent indole-3-acetic acid (IAA) production as well as that of the Ehrlich pathway for tryptophol production. We designated this *S. scitamineum* aminotransferase as SsAro8 and found that it was essential for mating/filamentation. Comparative metabolomics analysis revealed that SsAro8 was involved in tryptophan metabolism, likely for producing important intermediate products, including tryptophol. Exogenous addition of tryptophan or tryptophol could differentially restore mating/filamentation in the *ssaro8*Δ mutant, indicating that in addition to tryptophol, other product(s) of tryptophan catabolism may also be involved in *S. scitamineum* mating/filamentation regulation. *S. scitamineum* could also produce IAA, partially dependent on SsAro8 function. Surprisingly, photodestruction of IAA produced the compound(s) able to suppress *S. scitamineum* growth/differentiation. Lastly, we found that SsAro8 was required for proper biofilm formation, oxidative stress tolerance, and full pathogenicity in *S. scitamineum*. Overall, our study establishes the aminotransferase SsAro8 as an essential regulator of *S. scitamineum* pathogenic differentiation, as well as fungus-host interaction, and therefore of great potential as a molecular target for sugarcane smut disease control.

**IMPORTANCE** Sugarcane smut caused by the basidiomycete fungus *S. scitamineum* leads to massive economic losses in sugarcane plantation globally. Dikaryotic hyphae formation (filamentous growth) and biofilm formation are two important aspects in *S. scitamineum* pathogenesis, yet the molecular regulation of these two processes was not as extensively investigated as that in the model pathogenic fungi, e.g., Candida albicans, Ustilago maydis, or Cryptococcus neoformans. In this study, a tryptophan aminotransferase ortholog was identified in *S. scitamineum*, designated SsAro8. Functional characterization showed that SsAro8 positively regulates both filamentous growth and biofilm formation, respectively, via tryptophol-dependent and -independent manners. Furthermore, SsAro8 is required for full pathogenicity and, thus, is a promising molecular target for designing anti-smut strategy.

## INTRODUCTION

The sugarcane smut fungus Sporisorium scitamineum goes through three phases during its pathogenic life cycle, with distinct cell morphology and lifestyle, namely, haploid sporidium (yeast-like), dikaryotic hypha, and diploid teliospore. The sexual mating of two haploid sporidia of opposite mating types, *MAT-1* and *MAT-2*, results in formation of dikaryotic hyphae, which infect and grow within the host canes along with bud meristem and eventually form diploid teliospores by nuclear fusion. A round of meiosis occurs shortly afterwards and gives rise to four haploid sporidia (1). The molecular mechanism of sexual mating and filamentation is only limitedly reported in S. scitamineum, although it has been extensively investigated in the model smut fungus Ustilago maydis (2). The conserved mating loci, a and b loci, were identified and characterized, both of which were essential for *S. scitamineum* mating/filamentation (3, 4). The expression of a and b loci genes was under regulation of the pheromone response factor Prf1 (5), downstream of the cAMP-PKA signaling pathway (6) and the MAP kinase Kpp2 (7). Furthermore, redox signaling is involved in *S. scitamineum* mating/filamentation and virulence and subject to regulation of cAMP-PKA pathway (4, 6, 8). On the other hand, production of the fungal quorum-sensing (QS) signal, tryptophol, in a tryptophan-dependent manner, may be regulated by Kpp2 and the AGC kinase Agc1 to promote filamentation after sexual mating (7, 9).

The QS phenomenon was first reported and extensively studied in bacteria, where it is known to be involved in the regulation of various biological processes, including pathogenesis, symbiosis, competence, conjugation, nutrient uptake, morphological differentiation, secondary metabolite production, and biofilm formation (10). A lot of chemical molecules were identified as bacterial quorum-sensing molecules (QSMs) (11), but limited QSMs were identified in fungi (12). Fungal QS has been shown to regulate sporulation, secondary metabolite(s) production, morphological transition, and enzyme secretion (13). Examples include farnesol and three aromatic alcohols identified in Saccharomyces cerevisiae or Candida albicans, regulating yeast-to-hypha dimorphic switch, virulence, and/or pseudohyphae growth by integrating cell density and nitrogen availability (14, 15). α-(1, 3)-Glucan can regulate Histoplasma capsulate proliferation within a host macrophage (16). More functional QSMs await identification and characterization in plant-pathogenic fungi, including *S. scitamineum*.

The Ehrlich pathway reported in yeast is responsible for producing the aromatic alcohols by three steps: the deamination of the aromatic amino acids (tryptophan, tyrosine, and phenylalanine) by aromatic aminotransferases (AATs) to produce the corresponding α-keto acid analogues, decarboxylation by α-keto acid decarboxylases (KDCs) to aldehydes, and reduction by alcohol dehydrogenases (ADHs) to the corresponding aromatic alcohols (17, 18). Interestingly, the first two steps of the Ehrlich pathway are also used by plants and some microbes to produce the phytohormone indole-3-acetic acid (IAA), instead of tryptophol (TryOH), in a tryptophan-dependent manner (19, 20). In such a metabolism pathway, namely, the indole-3-pyruvic acid (IPA) pathway, tryptophan is deaminated to IPA, which is then decarboxylated to indole-3-acetaldehyde (IAAld) and then oxidized to IAA (21). At present, neither TryOH nor IAA biosynthesis pathway is identified in *S. scitamineum*. The biological function of these two metabolites in *S. scitamineum* growth, differentiation, and/or pathogenicity is also not fully understood.

In this study, we identified a tryptophan aminotransferase-encoding gene (*SSCI78970.1*) in *S. scitamineum*, named *SsARO8*. SsAro8 is involved in the fungal tryptophan catabolism pathway, contributing to TryOH and IAA production. TryOH was found to promote filamentous growth after sexual mating, likely via regulation of the MAPK and AGC signaling pathways established as essential for *S. scitamineum* mating/filamentation. On the other hand, IAA-derived compound(s) upon light induction displayed an inhibitory effect on *S. scitamineum* sporidial and filamentous growth. Furthermore, we found that SsAro8 is required for biofilm formation, oxidative stress tolerance, and full pathogenicity in *S. scitamineum*. Comparative transcriptome analysis was performed to further reveal the functional mechanism of SsAro8 in regulating *S. scitamineum* pathogenic development. Overall, our study establishes SsAro8 as a key enzyme in tryptophan metabolism, contributing to *S. scitamineum* pathogenicity.

## RESULTS

### Transcriptional regulation in response to aromatic alcohols.

**FIG 1:**
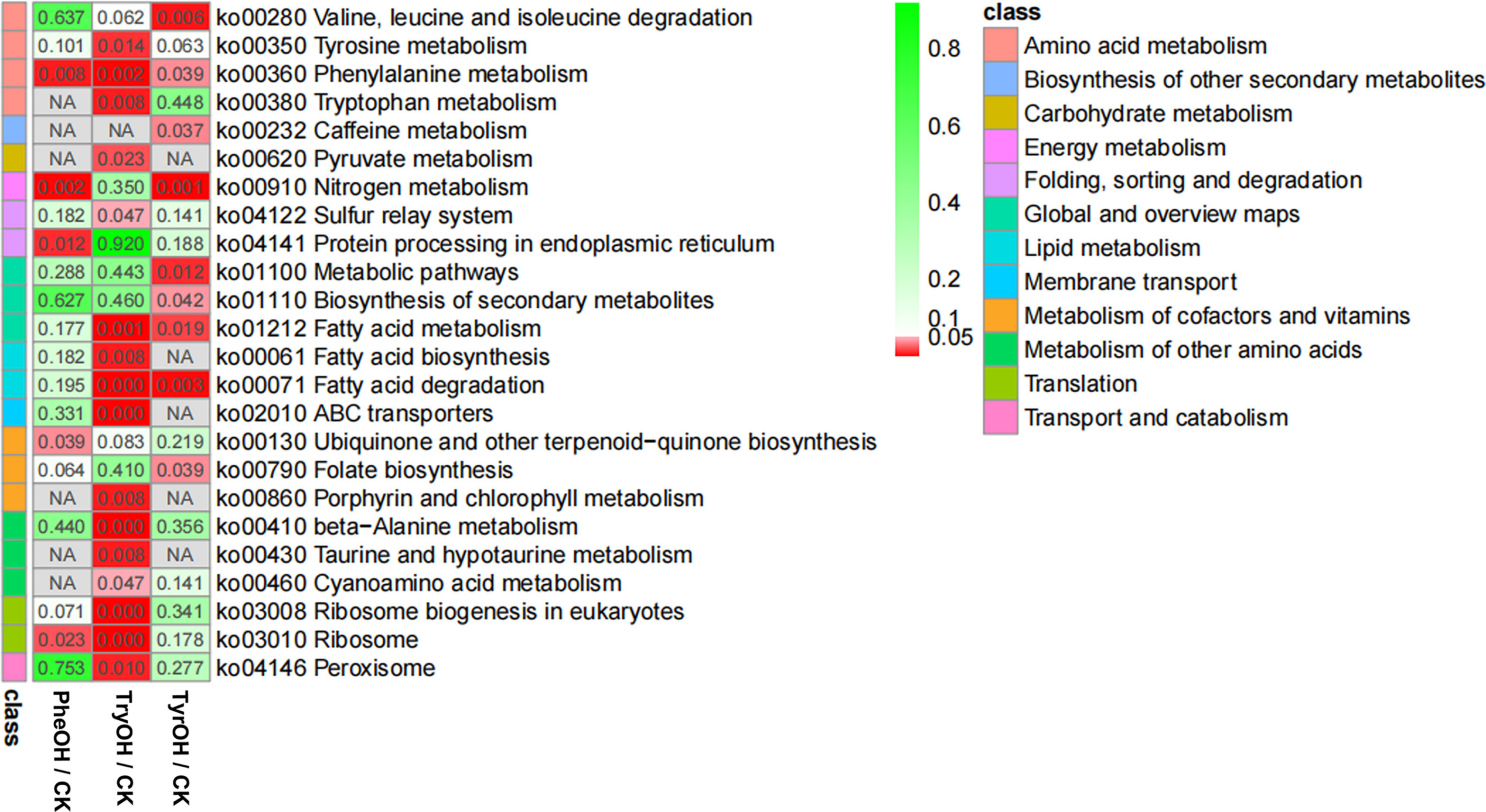
KEGG enrichment of differentially expressed genes in response to aromatic alcohols. Colored scale bars represent *P* values of each comparison and classes of KEGG pathways, respectively.

Three aromatic alcohols, tryptophol (TryOH), tyrosol (TyrOH), and phenylethyl alcohol (PheOH), have been reported as fungal QSMs to regulate dimorphic switch (12, 22, 23). Therefore, in this study, we used high-throughput RNA sequencing (RNA-Seq) analysis to screen for genes in response to the individual aromatic alcohol in the sugarcane smut fungus *S. scitamineum*, with the aim to identify the aromatic alcohol playing the major role in inducing *S. scitamineum* mating/filamentation. Our results (Data set S1) showed that 185 genes were differentially expressed (|log2FC| > 1 and false-discovery rate [FDR] of <0.05; three biological repeats) in the PheOH-treated sporidia, of which 140 were upregulated and 45 downregulated. TyrOH treatment caused 175 differentially expressed genes (DEGs), including 150 upregulated and 25 downregulated genes. We were interested to notice that TryOH treatment led to many more DEGs, a total of 1,299 (681 upregulated and 618 downregulated; Data set S1). GO and KEGG enrichment analysis demonstrates that PheOH-regulated genes are enriched in nitrogen metabolism, amino acid metabolism, and fatty acid metabolism pathways, and their encoding proteins are mostly localized on membrane and in nucleolus (Fig. S1; Data set S2 to S3). TyrOH-regulated genes are also involved in nitrogen metabolism, amino acid metabolism, and fatty acid metabolism. Furthermore, they are enriched in gene transcription and protein folding and therefore correspondingly distributed on transcriptional complexes and endoplasmic reticulum (ER) lumen (Fig. S2; Data set S2 to S3). TryOH-regulated expression of genes is responsible for carbon metabolism, amino acid metabolism, and fatty acid metabolism (Data set S2 to S3). The encoded proteins are largely localized on ribosomal complex, suggesting a major role in regulating protein synthesis (Fig. S3; Data set S2 to S3). TryOH-responsive genes are also enriched on peroxisome and ABC transporter, corresponding to their oxidoreductase activity and transporter activity (Fig. S3; Data set S2 to S3). Overall, all three aromatic alcohols regulate various metabolism processes and partially overlap each other, among which TryOH regulation of gene expression may be most predominant (Fig. 1). We were particularly interested to notice that TryOH could regulate metabolism of all the three aromatic amino acids, namely, phenylalanine, tyrosine, and tryptophan (Fig. 1), suggesting that it may affect production of the three aromatic alcohols, including itself. But in contrast, the other two aromatic alcohols could not affect tryptophan metabolism (Fig. 1) and thus are unlikely to affect TryOH production. Exogenous TryOH significantly induced expression of the pheromone receptor-encoding gene *PRA2* (Data set S1; Fig. S4) and *AGC1* gene (orthologous to yeast *RIM15*; Data set S1; Fig. S5), which was previously shown to positively regulate filamentous growth (9). Overall, our comparative transcriptome analysis reveals that *S. scitamineum* sporidia respond differentially to different aromatic alcohols, among which TryOH is specifically responsible for induction of mating/filamentous growth likely by integrating aromatic amino acid metabolism and signaling pathways.

### SsAro8 is essential for *S. scitamineum* mating/filamentation.

**FIG 2:**
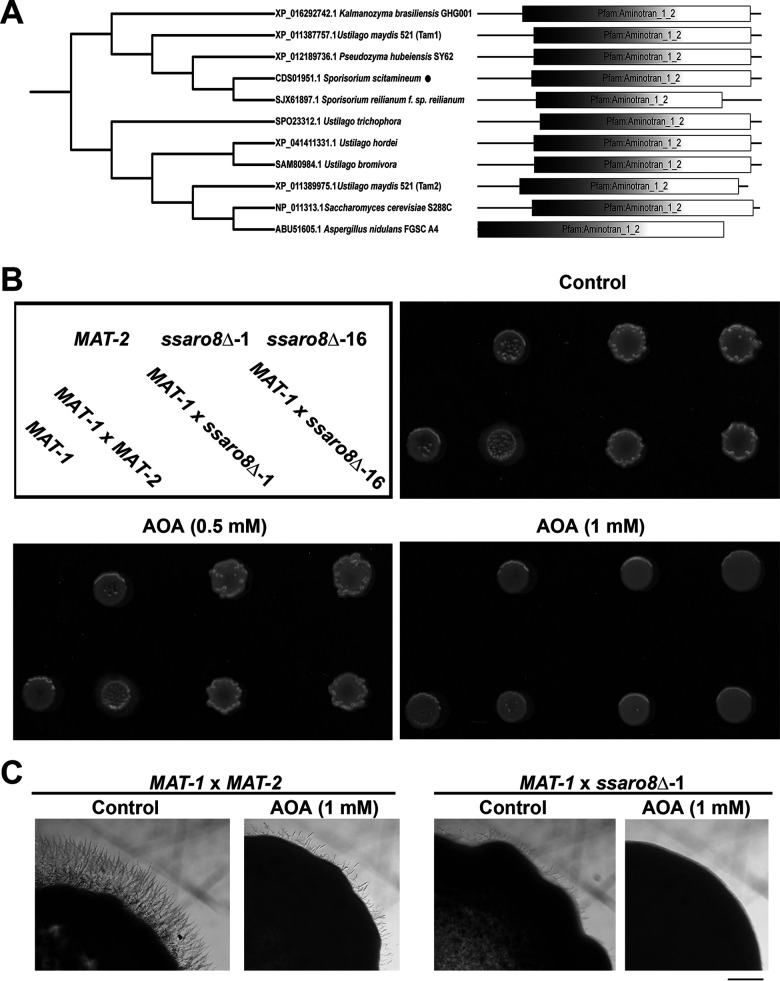
Identification and characterization of SsAro8. (A) Phylogenetic analysis of orthologous Aro8 proteins in yeast and fungi. Amino acid sequences were aligned using ClustalW 2.0 and analyzed by maximum likelihood methods with 1,000 bootstrap replications. Accession number for each protein used in this analysis was presented, followed by the corresponding species/strain name. *S. scitamineum* SsAro8 was denoted by a solid dot. Domain prediction was performed using SMART (http://smart.embl.de/). (B) Mating assay with the sporidia of wild-type *MAT-1*, respectively, with the wild-type *MAT-2*, *ssaro8*Δ-1, and -16 sporidia. The mixed sporidia of opposite mating type were inoculated on PDA, with or without AOA, and allowed to grow for 3 days before photographing. (C) Observation of fungal filamentation under stereomicroscopy, at 3 dpi. Bar = 1 mm.

We next investigated TryOH biosynthesis pathway in *S. scitamineum*. The yeast AAT Aro8 has been reported to catalyze deamination of aromatic amino acids, including tryptophan, as the first step of Ehrlich pathway to produce the corresponding aromatic alcohols (24–26). On the other hand, Ustilago maydis tryptophan aminotransferases Tam1 and Tam2 were reported responsible for tryptophan-dependent IAA biosynthesis, catalyzing deamination of tryptophan (27). Therefore, we searched for the *S. scitamineum* AAT ortholog by BLAST (Basic Local Alignment Search Tool, https://blast.ncbi.nlm.nih.gov/Blast.cgi) using S. cerevisiae Aro8 (KZV11027.1) and U. maydis Tam1 (XP_011387757.1) and Tam2 (XP_011389975.1) as the baits. We obtained a protein, CDS01951.1, displaying amino acid sequence similarity as 27.8% to Aro8, 89.66% to Tam1, and 55.79% to Tam2, respectively. Considering that we were looking for the AAT involved in biosynthesis of aromatic alcohol(s) rather than IAA, we named this protein SsAro8, following the naming of yeast Aro8. SsAro8 is encoded by the gene *SSCI78970.1* and is predicted as a peptide of 503 amino acids, containing a conserved Aminotran_1_2 domain (Fig. 2A). Phylogenetic analysis among yeast and filamentous/basidiomycetous fungi showed that SsAro8 is distantly related to S. cerevisiae Aro8 but more conserved in basidiomycetes, among which it is closely related to the annotated Sporisorium reilianum Aro8 (SJX61897.1; Fig. 2A). SsAro8 is also close to U. maydis Tam1 (XP_011387757.1) and relatively farther from U. maydis Tam2 (Fig. 2A). In addition to yeast Aro8, U. maydis Tam1 and Tam2 with established function in catalyzing tryptophan deamination (24, 27), SsAro8 is also close to ABU51605.1 (42.89% similarity), an aminotransferase proved to catalyze tryptophan deamination in Aspergillus nidulans (28). Overall, SsAro8 is close to several filamentous/basidiomycetous orthologs able to convert tryptophan to indole-3-pyruvic acid (IPA), which is a potential precursor for production of IAA, TryOH, or other indole metabolite(s).

To investigate the possible function of SsAro8 in *S. scitamineum* sexual mating and filamentous growth, we generated deletion mutants of *SsARO8* gene in *MAT-2* background. Schematic representation of gene deletion strategy and verification of gene deletion mutants were shown in Fig. S6A and B. The *MAT-1* wild-type sporidia were, respectively, mixed with wild-type or *ssaro8*Δ sporidia of *MAT-2* background and inoculated on peptone-dextrose agar (PDA) medium. Successful formation of dikaryotic hyphae was observed at 3 days postinoculation (dpi), as the appearance of white, fluffy colonies in wild-type *MAT-1*×*MAT-2* culture (Fig. 2B). However, *MAT-1*×*ssaro8*Δ exhibited obvious reduction in filamentous growth (Fig. 2B and C). We tested whether the defective filamentous growth in the *MAT-1*×*ssaro8*Δ culture was due to loss of AAT function by applying the AAT inhibitor aminooxyacetate (AOA) (29) to the *S. scitamineum* mating cultures. The result showed that AOA was effective in suppressing mating/filamentous growth of the wild-type *MAT-1*×*MAT-2* in a dose-dependent manner (Fig. 2B and C). We further verified the AOA inhibition on AAT function by measuring the endogenous level of IAA and TryOH in *S. scitamineum* sporidia, both of which are downstream products of IPA converted from tryptophan by AAT function (21). The result showed that IAA level was significantly reduced under AOA treatment (Fig. S6C and D), while TryOH was undetectable under experimental condition. This further indicates that the AAT function of SsAro8 is indeed critical for inducing *S. scitamineum* mating/filamentation.

### SsAro8 is involved in tryptophan metabolism pathway.

**FIG 3:**
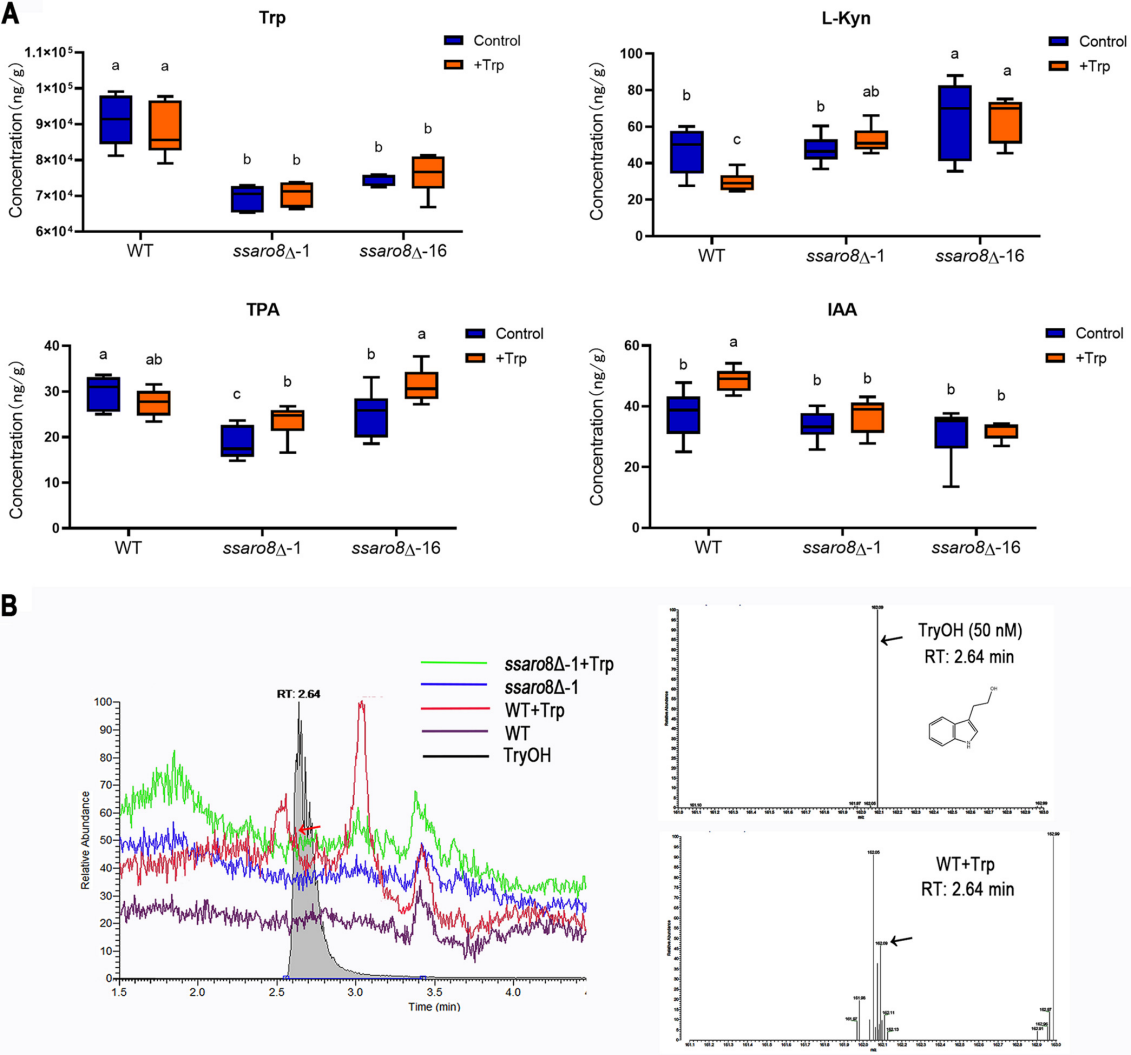
*SsARO8* is involved in tryptophan catabolism. (A) Contents for tryptophan (Trp), l-kyrunine (l-Kyn), tryptamine (TPA), and IAA of the wild-type (WT) or *ssaro8*Δ sporidia, cultured under control (untreated) or Trp-supplemented (+Trp) conditions, were quantified by tryptophan metabolomics analysis (details in Materials and Methods) and presented in boxplots. Different letters indicated significant difference (*P < *0.05, *n* = 6). (B) Detection of intracellular TryOH content from the WT or *ssaro8*Δ sporidia cultured under the same condition as that described in panel A. Fifty micromolar TryOH (Sigma, 188255) solution served as a standard. Left panel shows the HPLC chromatograms (10 μM injection) of TryOH (standard) or fungal extract(s). RT, retention time. Right panel, MS/MS spectra of the peak (RT = 2.64 min) in TryOH (standard) and WT+Trp sample, obtained by isolation of the protonated molecular ion (*m/z* 162.09). Arrows denote the peaks corresponding to TryOH.

Tryptophan could be catabolized mainly via indole pathways (including Ehrlich pathway and IPA pathway), kynurenine pathway, and serotonin pathway (29, 30). The yeast AAT Aro8 is able to catalyze tryptophan catabolism via either Ehrlich pathway or kynurenine pathway (31). Next, we further investigated the role of SsAro8 in tryptophan catabolism pathways by targeted metabolomics analysis using the wild-type (WT) and the *ssaro8*Δ sporidia under liquid culture condition, with or without supplementation of tryptophan (Trp) as the precursor. Our results showed that upon addition of Trp to the WT, IAA content was significantly increased while l-kynurenine (l-Kyn) content reduced significantly (Data set S4; Fig. 3A). Considering that l-Kyn was reported as an AAT inhibitor in plant (32, 33), such changes in IAA and l-Kyn contents suggest that the exogenously added Trp may be mainly catabolized via the indole pathways, including Trp-dependent IAA production pathways (34). On the other hand, products in serotonin pathway, including 5-hydroxy-l-tryptophan (5-HTP), serotonin, 5-hydroxyindole-3-acetic acid (5-HIAA), and melatonin, were either undetected or not much changed upon Trp addition (Data set S4), suggesting that serotonin pathway may not be responsible for Trp catabolism in *S. scitamineum*. Furthermore, both mutant strains (*ssaro8*Δ-1 and *ssaro8*Δ-16) displayed no changes in IAA contents when Trp was added (Data set S4; Fig. 3A). This result indicates that SsAro8 may be involved in Trp-dependent IAA production by converting Trp to IPA, as the reported IPA pathway (34). Under Trp supplement condition, IAA and its downstream product indole-3-carboxaldehyde were significantly reduced (Data set S4), while l-Kyn was significantly increased, in the *ssaro8*Δ mutants compared to that in the WT (Data set S4; Fig. 3A), confirming that SsAro8 is involved in deamination of Trp, loss of which may detour the exogenous Trp to kynurenine pathway for catabolism. IPA, the direct product of Aro8, is not stable and could not be detected in this study, due to technical limitation. IPA could be converted to indole-3-lactic acid (ILA), which was detected in all the samples, but indole-3-propionic acid (I3PA) downstream of ILA was not detected (Data set S4).

We also noticed the increased tryptamine (TPA) content in the *ssaro8*Δ mutants when Trp was added (Data set S4, Fig. 3A), indicating that Trp could be catabolized via an alternative IAA production pathway, TPA pathway (34). However, IAAld, produced by IPA, TPA, or TSO (tryptophan side chain oxidase) pathway and as the direct precursor of IAA (35, 36), was not detected in either WT or *ssaro8*Δ mutants (Data set S4). This suggests that these three IAA production pathways may not exist in *S. scitamineum*; alternatively, IAAld was quickly and completely metabolized to IAA so that it was not detected under our experimental condition. Given that the IPA pathway catalyzed by SsAro8 was indeed critical for IAA production, and exogenous Trp was quickly metabolized upon addition (Data set S4; Fig. 3A), we infer that IPA, TPA, and TSO pathways may exist in *S. scitamineum* and IPA pathway is a major pathway contributing to IAA production.

In addition to IAA, IAAld could also be converted to TryOH. We then performed liquid chromatography–mass spectrometry (LC-MS) analysis to measure the content of TryOH in the WT and *ssaro8*Δ sporidia, with or without Trp addition. Our result showed that TrpOH was detected only in Trp-supplemented WT strain (Fig. 3B and C), with a concentration calculated as approximately 1.6 nM based on the standard curve (Fig. S6C). Based on the intermediate products detected by the targeted metabolomics analysis, and our LC-MS analysis of TryOH, we infer that SsAro8 is involved in the IPA and Ehrlich pathways to produce IAA and TryOH, respectively, in *S. scitamineum*. When *SsARO8* is deleted, *S. scitamineum* may use alternative pathways, including TPA pathway and kynurenine pathway, for Trp catabolism. The proposed Trp catabolism pathways in *S. scitamineum* are summarized in Fig. S7A.

### Function of Trp metabolites and aromatic alcohols during *S. scitamineum* filamentous growth.

**FIG 4:**
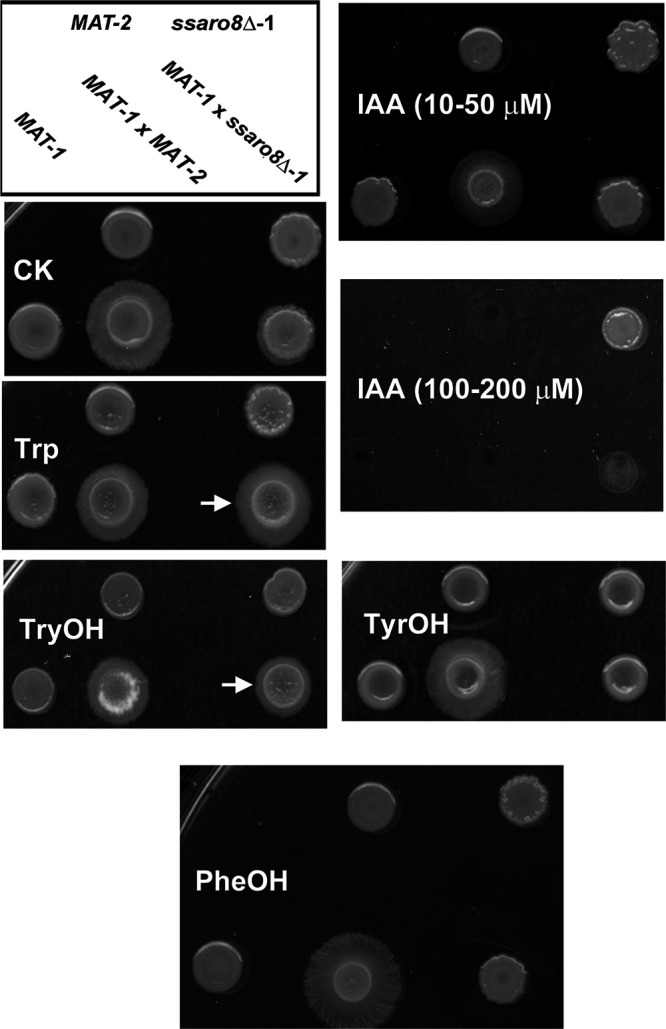
Effect of tryptophan metabolites or aromatic alcohols on *S. scitamineum* mating/filamentation. Mating/filamentation of WT and *ssaro8*Δ mutant was assessed on PDA medium, supplemented with the indicated chemical compounds. Photographs were taken at 3 dpi. Three biological repeats were performed for each instance, and representative images were displayed.

The metabolomics analysis showed that the *ssaro8*Δ mutants were compromised in Trp-dependent IAA and TryOH production, likely a reason for the defective mating/filamentous growth. To verify such, we supplemented the Trp, TryOH, and IAA, individually, to the mating culture of *MAT-1*×*ssaro8*Δ and tested their effect on filamentous growth. The result showed that exogenous supplementation of Trp (200 μM) or TryOH (10 to 30 μM) could significantly promote filamentous growth of *MAT-1*×*ssaro8*Δ (Fig. 4). However, higher concentration of TryOH (100 to 200 μM) suppressed filamentous growth of both *MAT-1*×*MAT-2* and *MAT-1*×*ssaro8*Δ cultures. On the other hand, IAA (10 to 50 μM) could not restore *MAT-1*×*ssaro8*Δ mating/filamentation and also suppressed sporidial and filamentous growth of the wild-type strain at a high concentration (100 to 200 μM; Fig. 4).

Considering that the yeast AAT Aro8 may also catalyze the deamination of the other two aromatic amino acids (tyrosine and phenylalanine) in the Ehrlich pathway (17), we also tested the effect of TyrOH and PheOH on *MAT-1*×*ssaro8*Δ filamentation. The result showed that they could not restore *MAT-1*×*ssaro8*Δ filamentation (Fig. 4) in the testing concentration range of 10 to 200 μM, therefore further confirming that SsAro8-catalyzed TryOH production promotes *S. scitamineum* mating/filamentous growth.

**FIG 5:**
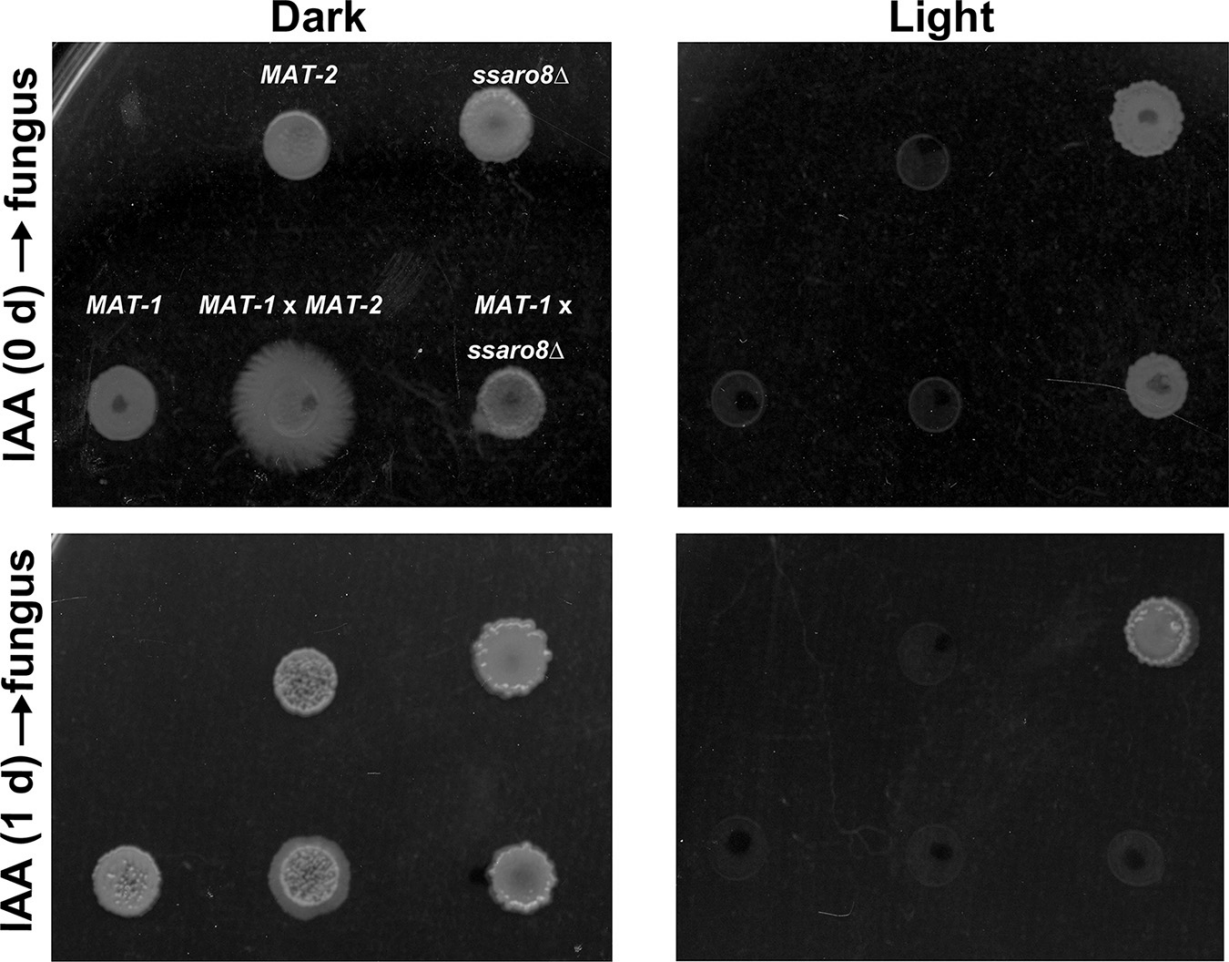
Light-induced toxic compound production derived from IAA. PDA containing 200 μM IAA was incubated under dark or light condition for 1 day, before sporidia (OD_600_ ≈ 1.0) or mating culture (1:1 mixed sporidia of opposite mating-type) were inoculated. The IAA-containing PDA without preincubation (0 days) was used as control. The inoculated PDA plates were kept under constant dark for 3 days before photographing. Three biological repeats were performed, and representative images are shown here.

We were interested to notice that the toxic effect of 200 μM IAA was more prominent when the fungal cells were cultured under light than when they were in the dark condition (Fig. 5). We infer that the high concentration of IAA could be further catabolized to produce a toxic compound, specifically under light. We also noticed that *ssaro8*Δ strain was less sensitive to IAA under light (Fig. 5) and inferred that it could be due to lower endogenous concentration of IAA in the *ssaro8*Δ mutant than in the wild-type strain. Furthermore, we found that the toxic compound(s) potentially derived from IAA could be formed without presence of live cells, as the sporidial and filamentous growth of *S. scitamineum* was suppressed when cells were cultured on the light-exposed (for 1 day before inoculating fungal cells) PDA medium containing 200 μM IAA but not when cells were cultured on the same medium kept in constant dark (Fig. 5). Therefore, we conclude that SsAro8-catalyzed Trp deamination is important for the following steps of the Ehrlich pathway for producing TryOH to promote *S. scitamineum* mating/filamentation. In contrast, production of IAA via IPA pathway may not be required for filamentous growth after sexual mating; instead, it may be further catabolized (without presence of live cells) to compounds toxic to *S. scitamineum*, especially under light condition. Overall, our study demonstrates that SsAro8 regulates *S. scitamineum* mating/filamentation likely via catalyzing TryOH production.

### Function of aromatic alcohols in *S. scitamineum* sporidial growth.

**FIG 6:**
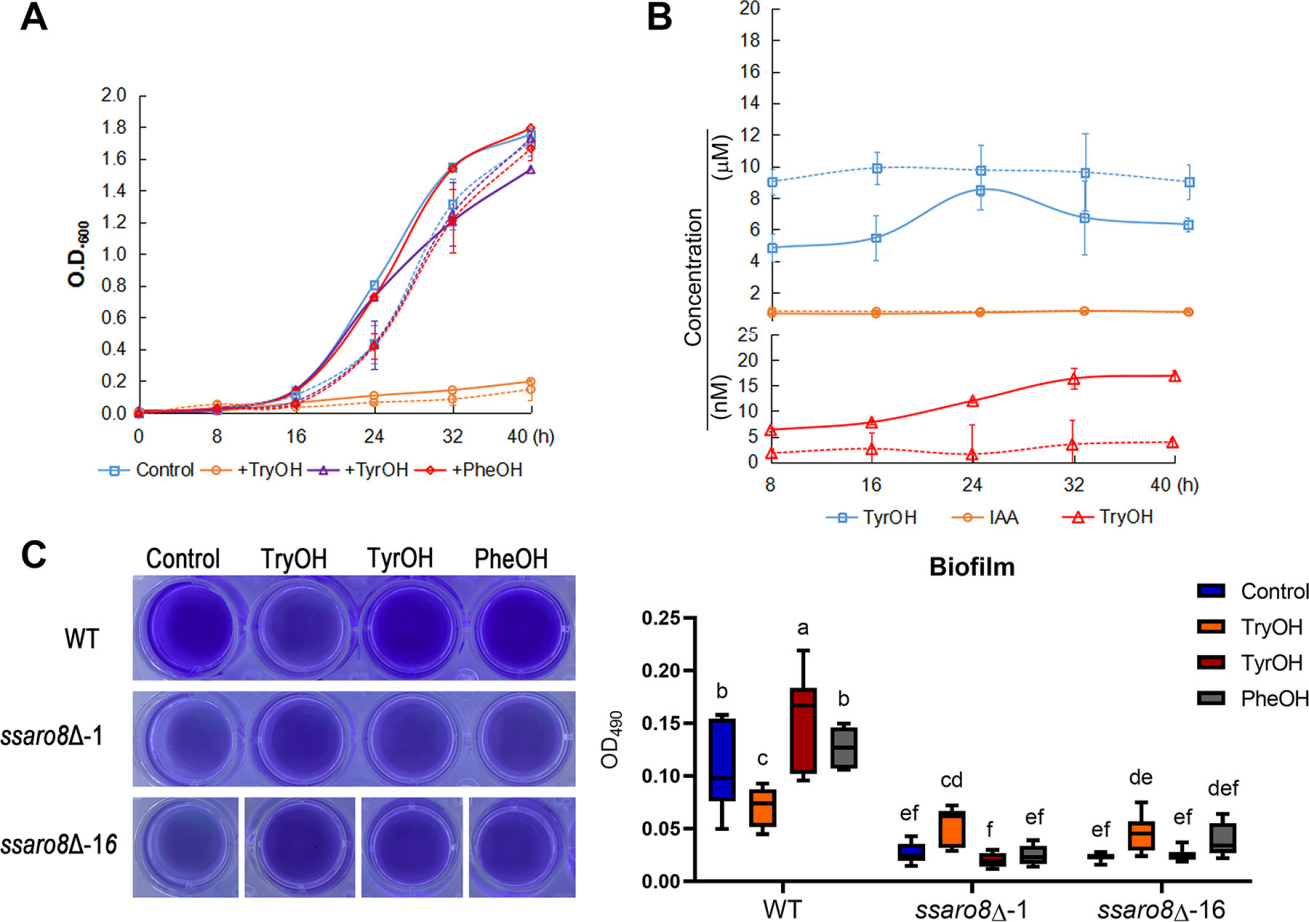
Effect of aromatic alcohols on *S. scitamineum* sporidial growth. (A) Growth curve of wild-type (solid lines) or *ssaro8*Δ (dashed lines) sporidia cultured in YePS medium, with or without supplement of TryOH (30 μM), TyrOH (200 μM), or PheOH (8 μM). Mean ± standard deviation (SD) was derived from three biological repeats. (B) Measurement of TyrOH, TryOH, or IAA in wild-type (solid lines) or *ssaro8*Δ (dashed lines) sporidia during time course. Mean ± SD was derived from three biological repeats. (C) Evaluation of biofilm formation by crystal violet staining. Wild-type and mutant cells were grown in 24-well dishes in YePS liquid medium (control) or supplemented with TryOH (30 μM), TyrOH (200 μM), or PheOH (8 μM), at 28°C, 200 rpm for 2 days, followed by static incubation at 28°C for another 4 days, before crystal violet staining and photographed. Representative images were shown from three biological repeats. Quantification of biofilm was performed based on OD_490_ absorption of the crystal violet-stained biofilms and represented as boxplots derived from three biological repeats. Different letters denote significant difference (*P < *0.05, *n* = 8).

We further investigated the role of aromatic alcohols in *S. scitamineum* sporidial growth by measuring the growth curve of the wild-type or *ssaro8*Δ sporidia cultured in the liquid YePS medium (yeast extraction 1%, peptone 2%, sugar 2% [pH 7.0]), with or without exogenous TyrOH, TryOH, or PheOH, respectively. We found that the *ssaro8*Δ mutant slightly lagged in sporidial growth compared to the wild type, at early stage, in either control (untreated) condition, or supplemented with individual aromatic alcohol (Fig. 6A). Exogenous supplement of PheOH did not change the growth curve of the wild-type or *ssaro8*Δ sporidia in comparison to that of the control condition, and supplement of TyrOH only marginally reduced the sporidial growth (Fig. 6A). In contrast, supplement of TryOH (30 μM) significantly suppressed sporidial growth in both wild-type and the *ssaro8*Δ strains (Fig. 6A). We infer that TryOH may be inhibitory to yeast-like growth, while promoting filamentous growth.

Furthermore, we measured the intracellular contents of TyrOH, IAA, and TryOH during such sporidial growth, in wild type and *ssaro8*Δ. We found that TyrOH content displayed a rhythmic oscillation in the wild-type sporidia during growth course (Fig. 6B). However, TyrOH in the *ssaro8*Δ was obviously higher than that in the wild-type sporidia and seemingly lost the rhythmic oscillation (Fig. 6B). IAA level showed no obvious difference between the wild-type and the *ssaro8*Δ sporidia (Fig. 6B). On the other hand, the TryOH level in the wild-type sporidia increased during growth course, in a cell density-dependent manner, while such trend was lost in the *ssaro8*Δ sporidia (Fig. 6B). This result suggests that SsAro8-mediated Trp deamination was used mainly for TryOH but not IAA production during sporidial growth.

### SsAro8 regulates *S. scitamineum* biofilm formation.

A biofilm is a surface-associated microbial community that can be defined as multicellular aggregates adhering to different surfaces and embedded within self-produced extracellular polymeric substances (EPS) (37, 38). Biofilm could be formed by bacteria or fungi and contributes to microbial resistance to antibiotics, as well as host infection in the case of pathogenic microbes (17, 38–42). We wonder if SsAro8 is involved in biofilm formation in *S. scitamineum*. Crystal violet staining was performed for visualization of biofilm formed by the wild-type or mutant sporidia, under different conditions. The result showed that biofilm formation was significantly reduced in *ssaro8*Δ mutants compared to that in the wild type (Fig. 6C). However, we were surprised to see that biofilm formation was reduced in the wild type supplemented with TryOH (30 μM), while slightly enhanced in the *ssaro8*Δ mutant under TryOH-supplement condition (Fig. 6C). The other two aromatic alcohols, TyrOH (200 μM) and PheOH (8 μM), did not affect biofilm formation in either wild type or *ssaro8*Δ (Fig. 6C). We infer that Trp catabolism mediated by SsAro8 may promote *S. scitamineum* biofilm formation, while TryOH suppresses biofilm formation, likely by suppressing yeast-like growth of sporidia.

### SsAro8 is important for tolerance to oxidative stress.

**FIG 7:**
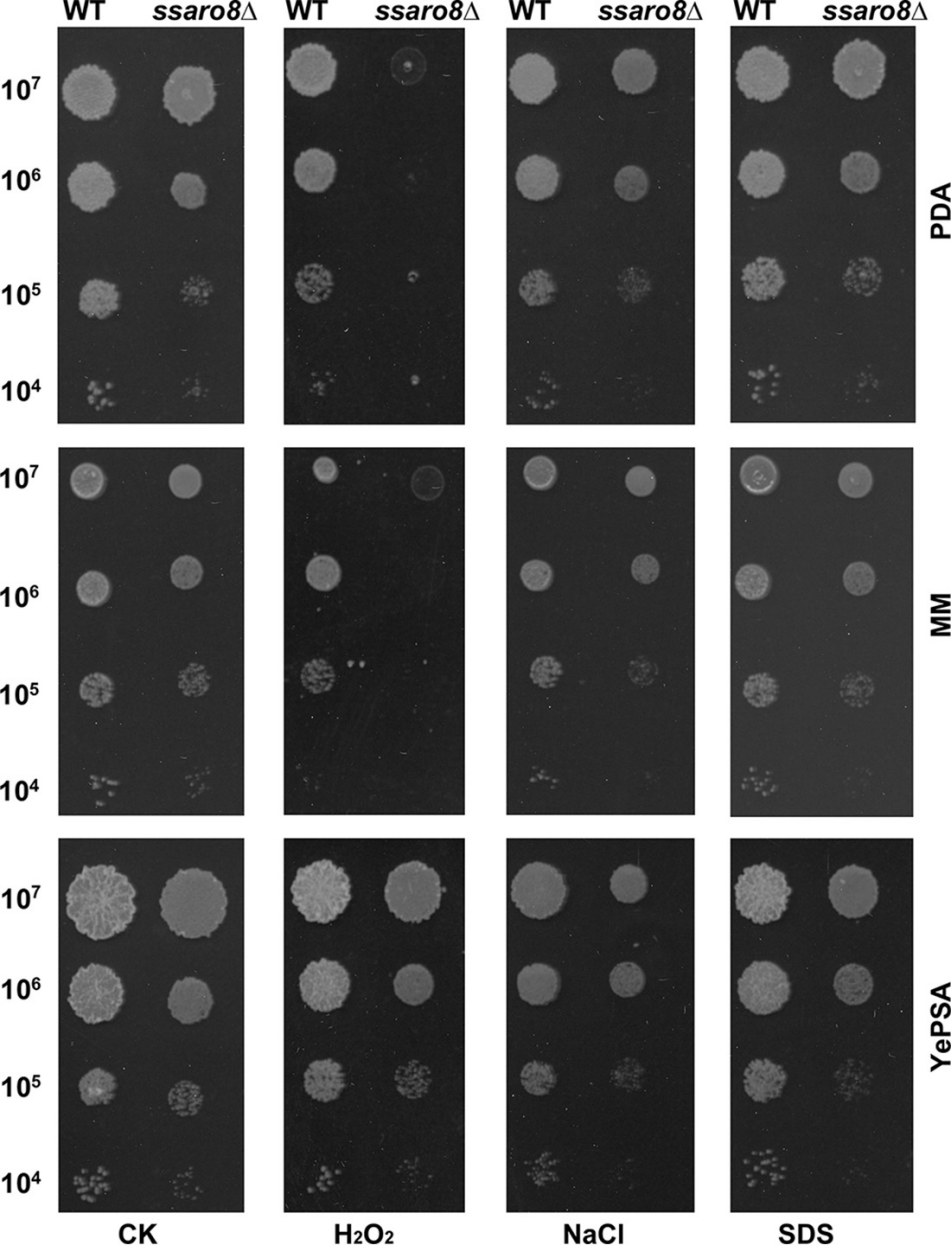
Evaluation of stress tolerance in the *ssaro8*Δ mutant. Serially diluted cells of *MAT-2* (WT) or *ssaro8*Δ mutant were spotted onto PDA, MM, or YePSA medium supplemented with H_2_O_2_ (1 mM), NaCl (500 mM), or SDS (0.1 mM). The control (CK) is the untreated culture. Images were taken at 3 dpi.

To evaluate the tolerance of *ssaro8*Δ mutant toward stressful conditions, we spotted the serially diluted mutant sporidia on solid medium supplemented with H_2_O_2_, NaCl, or SDS, along with the wild-type sporidia as a control. The colony morphology was assessed at 3 dpi. Results showed that *ssaro8*Δ exhibited an elevated sensitivity toward oxidative stress in comparison to that of the WT, particularly on PDA or minimal medium, but it was not obvious on YePSA (yeast extract 1%, peptone 2%, sugar 2%, agar 1.5%) (Fig. 7). Overall, we conclude that SsAro8 is involved in oxidative stress tolerance of *S. scitamineum*.

### Investigating functional mechanism of SsAro8 in regulating *S. scitamineum* sexual mating by transcriptome analysis.

As SsAro8 is a critical regulator of yeast-to-filamentation dimorphic switch in *S. scitamineum*, we further investigate the functional mechanism of SsAro8 by RNA-seq and comparative transcriptomic analysis. Wild-type (WT; *MAT-1*×*MAT-2*) and *ssaro8*Δ (*MAT-1*×*ssaro8*Δ) cultures were collected at 0 h (unmating) and 12 h (early stage of sexual mating), respectively. DEGs (|log2FC| > 1 and FDR of <0.05; three biological repeats) in different comparison were as listed in Data set S5. In total, 1,733 DEGs (1,098 upregulated and 635 downregulated) were identified in WT at 12 h versus 0 h (Data set S5), potentially as genes involved in *S. scitamineum* sexual mating. However, in the *ssaro8*Δ mating samples, 2,034 DEGs (1,260 upregulated and 774 downregulated) were identified, 418 of which did not overlap the DEGs in WT (12 h versus 0 h), suggesting that SsAro8 may be required for transcriptional regulation of these overlapped genes (Data set S5). Furthermore, we compared these 418 genes to the DEGs of *ssaro8*Δ versus WT, at 12 h, and found that 60 genes overlapped (Data set S5). We infer that these 60 genes are dependent on SsAro8 for differential regulation at 12 h, a critical stage of *S. scitamineum* sexual mating. We further found 24 overlapped genes between these 60 genes and TryOH-regulated genes (Data set S1; Data set S5, genes in red font in sheet named “Ssaro8-dependent DEGs”). The *a* locus gene *PRA2* is in this category (Data set S5, genes in red font in sheet named “Ssaro8-dependent DEGs”), confirming that SsAro8-mediated TryOH biosynthesis is indeed critical for *S. scitamineum* sexual mating.

On the other hand, we noticed that the tryptophan metabolism pathway was differentially regulated in *ssaro8*Δ at 12 h compared to that in WT (Fig. S8). These DEGs include *SsARO8* itself (log2FC of −2.469173815, FDR of 0; Data set S5) and upregulated *SsARO9* (*SPSC_00463*, log2FC of 1.033853611, FDR of 8.48E−72; Data set S5) likely as a compensation for loss of *SsARO8* in *MAT-2* background. An annotated aldehyde dehydrogenase (ALDH)-encoding gene (*SPSC_00448*) was also upregulated (log2FC of 1.719145151, FDR of 4.42E−43; Data set S5). We infer that loss of SsAro8 function may be at least partially compensated by upregulation of *SsARO9* gene, which also encodes an AAT, and meanwhile, the upregulation of the downstream ALDH-encoding gene may promote conversion from IAAld to IAA, instead of TryOH, resulting in reduction of intracellular TryOH but not much change in IAA content (Fig. 3B and 6B). Overall, the comparative transcriptomic analysis further supports that SsAro8 plays an important role in tryptophan catabolism during *S. scitamineum* dimorphic switch.

### SsAro8 is important for *S. scitamineum* full pathogenicity.

**FIG 8:**
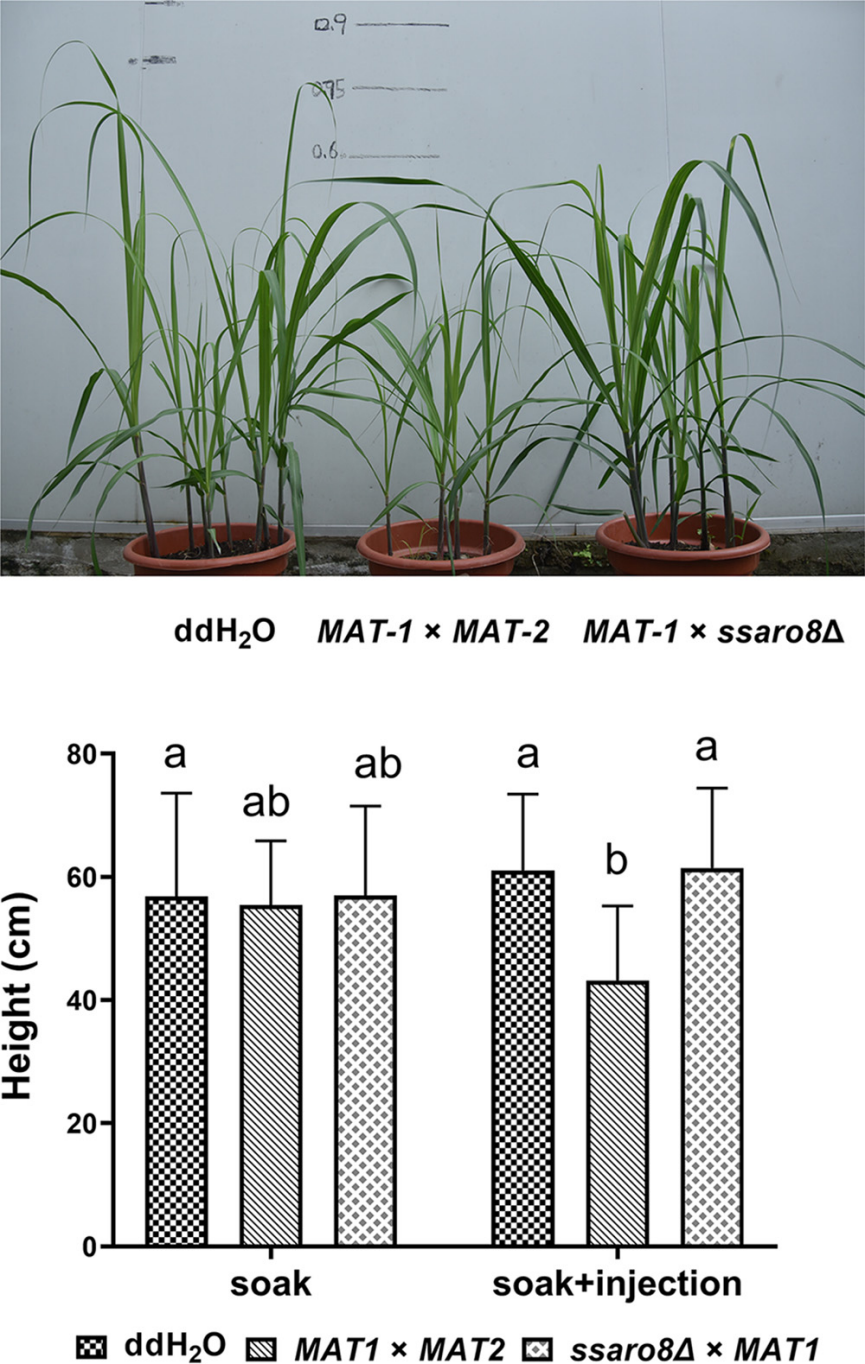
Pathogenicity assay. Images showing sugarcane seedings infected by *MAT-1*×*MAT-2* or *MAT-1*×*ssaro8*Δ mixed sporidia, using soaking plus injection method. ddH_2_O served as the blank control. Seedling heights were quantified and depicted in the bar chart. Mean ± standard error (SE) was derived from two repeats, each containing approximately 20 seedlings for each treatment. Photographing and seedling height measurement were performed at the 3rd month after sugarcane planting.

Given that SsAro8 is important for *S. scitamineum* filamentous growth, biofilm formation, and oxidative stress tolerance, we speculate that it may also be involved in pathogenicity. Therefore, we performed pathogenicity assay with WT (*MAT-1*×*MAT-2*) and *ssaro8*Δ (*MAT-1*×*ssaro8*Δ) sporidial mixed cultures. The stem sections of the highly susceptible sugarcane cultivar, ROC22, were soaked in the WT or mutant sporidial mixture for 1 day (kept under 28°C) before being planted in the pots. Such soaking-inoculated seedlings were allowed to grow for approximately 3 months until symptom examination. A portion of the seedlings were injected with the WT or mutant mixed sporidia at 2 weeks post planting. Although neither WT- nor mutant-infected seedlings show typical “whip” symptom at the 3rd month post planting, we did observe an obvious dwarf symptom of the WT-infected seedlings compared to *MAT-1*×*ssaro8*Δ-infected seedlings or the negative control (double-distilled water [ddH_2_O] inoculation) (Fig. 8). This result indicates that SsAro8 is required for causing seedling dwarf symptom. It remains to be confirmed whether SsAro8 is required for whip symptom development.

In summary, our study identified an orthologous Aro8 protein in *S. scitamineum* responsible for Trp-based TryOH and IAA production, which is crucial for fungal growth and pathogenic differentiation.

## DISCUSSION

In yeast and C. albicans, aromatic alcohols have been shown as important signaling molecules in regulating dimorphic switch by integrating cell density and nutrient status (43–46). Our previous study reported that an AGC kinase regulates *S. scitamineum* mating/filamentation in response to TryOH, a potential QSM in *S. scitamineum* (9). In this study, our transcriptome analysis confirmed that the genes controlling *S. scitamineum* mating/filamentation, including *a* locus gene *PRA2* and the aforementioned AGC kinase, were indeed induced by TryOH but not by the other two aromatic alcohols (TyrOH and PheOH). Furthermore, the transcriptome analysis indicates that TryOH regulates production of the other two aromatic alcohols but not *vice versa*. We infer that TryOH plays a predominant role in controlling *S. scitamineum* mating/filamentation, likely integrating signaling pathways and aromatic amino acids metabolism. To further elucidate the biosynthesis pathway of TryOH, we identified the Tam1/Tam2/Aro8 ortholog, named SsAro8 in *S. scitamineum*, and by targeted metabolomics and LC-MS analyses we confirmed that it was indeed involved in Trp metabolism, contributing to TryOH and IAA production. Loss of *SsARO8* led to defective mating/filamentation, which could be fully restored by exogenous addition of Trp and partially restored by addition of TryOH. This result, combined with the metabolomics analysis, indicates that SsAro8 is not only involved in Trp-dependent TryOH production but may also be required for synthesis of Trp. Furthermore, other catabolite(s) of Trp may also contribute to *S. scitamineum* mating/filamentation. Interestingly, the transcriptome analysis showed that *SsARO8* gene was significantly downregulated in response to exogenously added TryOH (Data set S1; Fig. S7B), indicative of a possible negative feedback in autoregulation of Trp-dependent TryOH production.

In addition to TryOH, *S. scitamineum* also produces the phytohormone IAA, likely using SsAro8 for the first step (tryptophan deamination) of the IPA pathway. In this study, we observed that exogenous IAA could be converted to some compounds toxic to *S. scitamineum* when exposed to light, without presence of live cells, suggesting that such conversion is a nonenzymatic reaction. A photooxidation of IAA has been reported (47, 48), forming products including 3-hydroxymethyl oxindole (HMO) and 3-methylene oxindole (Meox) (49). It would be of great interest to verify whether these two substances are responsible for growth suppression activity of high-concentration IAA to *S. scitamineum*, especially after exposure to light. IAA is the active form of the phytohormone auxin and known as an important regulator of plant growth and development, as well as plant-microbe, including plant-fungus, interaction (34). Another smut fungus, S. reilianum, can cause a range of morphological changes in the infected host maize, likely via manipulating host IAA biosynthesis and/or transport (50, 51). Similarly, whip formation caused by *S. scitamineum* infection to the host cane is related to altered auxin transport and signaling in plant (52). In this study, it draws our attention that *S. scitamineum* could also produce IAA, while the photodestruction of IAA produces the compound(s) able to suppress *S. scitamineum* growth/differentiation. This adds a new level of complication in the plant-fungus interaction, as both plant and the pathogenic fungus produce IAA, and such IAA-mediated cross-kingdom communication may be subject to regulation by environmental cues, for example, light.

Fungus-derived IAA was also reported in the smut fungus U. maydis, which could induce the rise in host IAA levels during fungal infection but is dispensable for host tumor formation (27, 53). IPA pathway is used by U. maydis for IAA production in glucose-containing medium, with the AATs Tam1 and Tam2 catalyzing the step of tryptophan deamination (27). SsAro8 is orthologous to U. maydis Tam1, supporting a functional conservation between these two smut fungi in terms of IAA production. However, in U. maydis, it has not been reported whether TryOH is produced or serves any biological function(s), neither do we know whether U. maydis Tam1 is also required for TryOH biosynthesis.

We also notice the rhythmic oscillation of TyrOH in the wild-type sporidia during growth course. TyrOH is another aromatic alcohol and a reported fungal QSM (46, 54). Deletion of *SsARO8* led to loss of such rhythmic oscillation but instead a constant higher level of intracellular TyrOH. Therefore, we infer that TyrOH production may be indirectly regulated by SsAro8 function, through SsAro8-catalyzed TryOH production. The comparative transcriptome analysis supports this hypothesis, as TryOH-regulated genes are enriched in tyrosine metabolism (Fig. 1). However, changed TyrOH content in *ssaro8*Δ mutant is not responsible for the defects in mating/filamentation but may be of biological significance in other aspect(s), awaiting further elucidation in future.

In this study, we also investigated the potential function of SsAro8 and TryOH in *S. scitamineum* biofilm formation. Intriguingly, high-concentration TryOH suppressed biofilm formation from the wild-type sporidia, while TyrOH or PheOH displayed no obvious effect (Fig. 6C). Unexpectedly, the *ssaro8*Δ mutant, with intracellular TryOH content lower than that of the wild-type sporidia (Fig. 6B), was also defective in biofilm formation (Fig. 6C). We infer that SsAro8 positively regulates *S. scitamineum* biofilm formation via a TryOH-independent mechanism. The defective biofilm formation in the *ssaro8*Δ mutant may be due to the delayed sporidial growth (Fig. 6A). Alternatively, deletion of *SsARO8* gene may result in altered exopolysaccharide (EPS) composition, an important component of the biofilm, due to the disrupted metabolism of aromatic amino acids, as reported in S. cerevisiae (17). On the other hand, our transcriptome analysis showed that TryOH treatment downregulated the potential positive regulator of biofilm formation, *SsARO8* gene, while upregulating the genes encoding negative regulators of the known fungal biofilm formation, including *TEC1*, *EFG1*, and *ROB1* (38) (Data set S1; Fig. S7B). This result provides a possible explanation to the inhibitory effect of TryOH on *S. scitamineum* biofilm formation. On the other hand, expression of these biofilm formation negative regulator-encoding genes was not significantly changed in the *ssaro8*Δ compared to that in the WT (Data set S5), suggesting that the reduced biofilm formation in the *ssaro8*Δ mutant may be due to slower yeast-like growth. Overall, we conclude that TryOH negatively regulates *S. scitamineum* biofilm formation, while SsAro8 affects biofilm formation, likely in an indirect way.

Transcriptome analysis between WT and *ssaro8*Δ during dimorphic switch further confirms that SsAro8-catalyzed TryOH plays an important role in inducing *S. scitamineum* sexual mating, likely via induction of the pheromone receptor-encoding gene, *PRA2*, of *MAT-2* background (Data set S5). We shortlisted 24 overlapped genes between 60 potential SsAro8-dependent DEGs during sexual mating (12 h) and TryOH-regulated genes (Data set S5), among which we notice several genes encoding signaling transducers, including a histidine kinase (SPSC_01045), a leucine-rich repeat-containing (LRR) protein (SPSC_03533), and a protein kinase (SPSC_03693) (Data set S5). These 24 shortlisted genes, particularly the signal transducer encoding genes, are worth further investigating in future to elucidate molecular mechanisms underlying QS-regulation of *S. scitamineum* dimorphic switch via SsAro8-mediated TryOH production.

In summary, this study showed that Trp catabolism via indole pathways plays an important role in regulating growth and pathogenic differentiation of the sugarcane smut fungus *S. scitamineum* by producing the phytohormone IAA and a potential fungal QSM TryOH. SsAro8 acts at a common step upstream of both IAA and TryOH biosynthesis pathways and thus potentially functions in balancing fungal differentiation and interaction with the host plant. Furthermore, SsAro8 regulates the key pathogenic processes of *S. scitamineum*, including dikaryotic hyphae formation (filamentous growth) and biofilm formation, and thus can potentially be used as an antifungal target for sugarcane smut disease control.

## MATERIALS AND METHODS

### Growth conditions and fungal strains used in this study.

Teliospores of sugarcane smut collected from the fields in Guangdong province of China (21°12′ 36″ N; 101°10′ 12″ E) by Yan (3) were maintained in Yi Zhen Deng’s lab, and the *MAT-1* and *MAT-2* haploid sporidia isolated from such teliospores were used in this study. The culture medium used in this study include YePSA medium (yeast extract 1%, peptone 2%, sugar 2%, agar 1.5%), YePS liquid medium (yeast extraction 1%, peptone 2%, sugar 2% [pH 7.0]), and PDA medium (PDA powder 4%, from DingGuo, Guangzhou, China). For mating/filamentation assay, equal volumes of haploid sporidia (optical density at 600 nm [OD_600_] ≈ 1.0) of opposite mating types were mixed, plated on the PDA medium, and kept in dark at 28°C incubator for 2 to 3 days before photographing.

For assessment of sporidial budding, 1 mL of the wild-type or *ssaro8*Δ sporidia (OD_600_ ≈ 1.0) was added in 500 mL YePS liquid medium and then cultured at 28°C, 200 rpm for 2 days. During this course, 11 mL of such sporidial culture was collected every 4 h, 1 mL of which was used for measuring the OD absorption at 600 nm to determine the sporidial cell number and the remaining 10 mL of which was used for ethyl acetate extraction followed by high-resolution electrospray ionization mass spectrometry (HR-ESI-MS) analysis to determine the concentration of TyrOH, TryOH, and IAA. This experiment was repeated three times, each containing three biological repeats.

For biofilm formation, the haploid sporidia (OD_600 _≈ 1.0) were diluted 100-folds with YePS, added to 24-well plates (1 mL per well), and then cultured at 28°C with shaking (200 rpm) for 2 days, followed by static incubation at 28°C for another 4 days, before crystal violet staining and photographed. The cultured cells were washed with 1 mL sterilized water before staining with 1 mL 0.1% (wt/vol) crystal violet at room temperature for 20 to 30 min. The stained cells were then washed with sterilized water 3 times and dried. One milliliter of ethanol absolute was used for each well to dissolve the biofilm, and OD_490_ absorption was measured as a readout of biofilm formation, with ethanol absolute as the blank control. Three biological repeats were performed.

For stress tolerance assay, haploid sporidia (OD_600_ ≈ 1.0, equivalent to CFU ≈ 10^7^) were diluted in a gradient into 10^7^, 10^6^, 10^5^, and 10^4^ (CFU), spotted onto the solid medium, supplemented with H_2_O_2_ (1 mM), NaCl (400 mM), or SDS (0.1 mM), and kept in dark in a 28°C incubator for 2 to 3 days before photographing.

### Chemical compounds used in this study.

Amino-oxyacetic acid (AOA; Aldrich, C13408), tryptophan (Trp; Sigma, T0254), tryptophol (TryOH; Sigma-Aldrich, V900672), indole-3-acetic acid (IAA; Sigma, I2886), tyrosol (TyrOH; Sigma, 188255), and phenylethyl alcohol (PheOH; Sigma, P103660) were used in this study.

### Nucleic acid related manipulation.

Genomic DNA was extracted from sporidia using the fungal DNA midi kit (Omega, D3590-01). The resultant genomic DNA was used for Southern blotting hybridization, with the digoxigenin (DIG) high prime DNA labeling and detection starter kit II (Roche, Mannheim, Germany). Two micrograms of genomic DNA of the wild-type strain and transformants were digested with EcoRI/SacI-HF (NEB). Digested products were resolved in a 1% agarose gel and then transferred onto hybond-N1 membrane (Amersham). The 5′ flank sequence of *SsARO8* was used for PCR amplification of the specific probe, which was labeled with digoxigenin-11-dUTP using digoxigenin (DIG)-high prime, and then hybridization and detection were performed according to the instruction manual (Roche Applied Science).

For comparative transcriptome analysis, WT sporidia were grown in the liquid YePS medium, with or without supplement of individual aromatic alcohol, TryOH (30 μM), TyrOH (200 μM), or PheOH (8 μM), for 48 h before total RNA extraction using the Qiagen RNAeasy minikit (74104). For comparison between WT and *ssaro8*Δ mutant during sexual mating, WT (*MAT-1*×*MAT-2*) and *ssaro8*Δ (*MAT-1*×*ssaro8*Δ) cultures were collected at 0 h (unmating) and 12 h (early stage of sexual mating), respectively, for total RNA extraction using the Qiagen RNAeasy mini kit (74104). Three biological replicates were performed for each treatment. High-throughput RNA sequencing (RNA-Seq) and transcriptome analysis were performed by Gene Denovo Co. (Guangzhou, China). Short reads were mapped to the complete genome of *S. scitamineum* (GenBank: GCA_900002365.1) using Tophat (55). Genes with ∣log2fc∣ ≥ 1 and false-discovery rate(FDR) ≤ 0.05 in a comparison were considered as differentially expressed genes (DEGs), and subject to enrichment analysis of Gene Ontology (GO) functions and KEGG pathways, following established protocols (56).

### Construction of *ssaro8*Δ mutant.

Double-joint PCR was performed to construct the fragment for the replacement of *SsARO8* gene by the *HPT* (Hyg^r^) gene following the strategy described previously (57, 58). The flanking DNA (1 kb 5′and 3′) of the *SsARO8* gene was PCR amplified using genomic DNA of *S. scitamineum MAT-2* strain as the template and the *HPT* gene with plasmid pEX2 (9) as the template. The primers were as listed in Table S1. The fragment containing the *HPT* gene franked by the 5′- and 3′-flanking sequences of *SsARO8* was verified by sequencing before being transformed into *MAT-2* protoplasts via polyethylene glycol (PEG) mediate protoplast transformation, following the established protocol (7). Lysing enzyme (Sigma, L1412) was dissolved in SCS solution (20 mM trisodium citrate and 1 M d-sorbitol [pH 5.8]) to reach the final concentration of 10 mg/mL and used for enzyme digestion of fungal cell wall to generate protoplasts. Forty percent PEG (Sigma-Aldrich, 202444) solution was prepared in 10 mL STC solution (10 mM Tris–HCl [pH 7.5], 1 M d-sorbitol, and 100 mM CaCl_2_). One to five micrograms of the PCR-amplified fragment was mixed with 1 μL heparin solution (15 mg/mL; Dingguo, DH157) and the protoplasts and incubated with 40% PEG solution on ice for 10 min. The protoplasts were regenerated on the 3-layer regeneration medium composed of the top layer of YePS soft medium (YePSA with 0.75% agar) plus two layers of YePSS medium (YePSA with 1M d-sorbitol), with only the bottom YePSS layer containing 400 μg/mL hygromycin B (Calbiochem, CAS:53-84-9) for primary screening of transformants based on antibiotic resistance. The *ssaro8*Δ mutants were further verified by Southern blotting.

### Determination of TyrOH, TryOH, and IAA concentration in sporidial culture.

A 1.5× volume of ethyl acetate was added to the wild-type or *ssaro8*Δ sporidia collected every 4 h during the 2 days’ time course and dried by rotary evaporation (EYELA, OSB-2100). The crude extracts were then dissolved in methanol and filtered with 0.22-μm polyvinylidene difluoride (PVDF) membrane (Nylon6) before HESI-MS analysis to determine the concentration of TyrOH, TryOH, or IAA.

The LC-MS method was performed on a Dionex UltiMate 3000 system (Thermo Fisher Scientific) using a C_18_ reverse-phase column (Thermo Fisher Scientific) and various concentration gradients of methanol and consisted of 0.1% acidified water as mobile phase. The gradient profile for chromatography was as follows: liner decrease in methanol from 95% to 5% over 4 min, keep the methanol 5% for 1 min, and immediately increase to 95% in a few seconds; keep the methanol 95% until the method ends at 7 min. The flow rate was constant at 0.3 mL/min.

Compounds were detected by heated electrospray ionization coupled to high-resolution mass spectroscopy (HESI-MS; Q Exactive Focus, Thermo Fisher Scientific). The analysis was performed under positive ionization mode. Settings for the ion source were 10 aux gas flow rate, 40 sheath gas flow rate, 0 sweep gas flow rate, 4 kV spray voltage, 320°C capillary temperature, 350°C heater temperature, and 50 S-lens RF level. Nitrogen was used as a nebulizing gas by the ion trap source. The tandem mass spectrometry (MS/MS) method was designed to perform an MS1 full-scan (100 to 1,000 *m*/*z*, no fragmentation) together with the SIM model. Settings for the SIM method were 35,000 resolution, 1.0 *m/z* isolation offset, 4.0 isolation window, and centroid spectrum. Signal mass spectrometry scans were set to C10H11NO at 162.09134 *m/z*, C8H10O2 at 139.07536 *m/z*, and C10H9N02 at 176.07061 *m/z*. Data analysis was performed using the Thermo Xcalibur software (Thermo Fisher Scientific).

### Tryptophan metabolomics analysis.

The 10 μL of wild-type or mutant sporidia (OD_600 _≈ 1.0) was diluted into 100 mL YnPS medium (10g/L yeast nitrogen base [YNB], 20g/L peptone, 20g/L sucrose), with or without supplementation of 1 mg/mL l-tryptophan (Trp), and allowed to grow at 28°C, 200 rpm for 2 days. The fungal samples were collected by centrifugation and sent for targeted metabolomics analysis of tryptophan metabolites by Shanghai Applied Protein Technology Co. Ltd. The collected fungal samples were weighted (100 ± 5 mg per sample) and homogenized using Biospec MiniBeadbeater24 (Fastprep24). Ten microliters of internal standard solution and 0.5 mL water-acetonitrile-methanol (1:2:2, vol/vol/vol) solution were added into the homogenized samples. Four hundred microliters of supernatant from the samples was collected and dried under nitrogen gas after centrifugation. The residue was redissolved in 100 μL acetonitrile-water (1:1, vol/vol) solution and then centrifuged at 14,000 × *g*. The supernatant was injected for high-pressure liquid chromatography–tandem mass spectrometry (HPLC-MS/MS) analysis.

The separation was performed on an ultraperformance liquid chromatography (UPLC) system (Agilent 1290 Infinity UHPLC) using a C_18_ column (Waters, CSH C_18_ 1.7 μm, 2.1 mm by 100 mm column) by gradient elution. Eluents A and B were acetonitrile and water consisting of 20 mM ammonium formate buffer (pH 3.7), respectively. The gradient elution program was as follows: 0 min, 15% B, 2 min, 15% B, 9 min, 98% B, 11 min, 98% B, 11.5 min, 15% B, and 14 min, 15% B. Before injecting the next sample, the column was equilibrated with the initial mobile phase for 5 min. The flow rate was 0.4 mL/min and the column temperature was set at 50°C. 5500 QTRAP (AB SCIEX) was performed in positive and negative switch mode. The ESI positive source conditions were as follows: source temperature, 550°C; ion source gas 1 (Gas1), 55; ion source gas 2 (Gas2), 55; curtain gas (CUR), 40; ionSapary voltage floating (ISVF), +4,500 V. The ESI negative source conditions were as follows: source temperature, 550°C; ion source gas 1 (Gas1), 55; ion source gas 2 (Gas2), 55; curtain gas (CUR), 40; ionSapary voltage floating (ISVF), −4,500 V. Multiple reaction monitoring (MRM) method was used for mass spectrometry quantitative data acquisition. The APCI source conditions were as follows: source temperature, 550°C; ion source gas 1 (Gas1), 55; ion source gas 2 (Gas2), 55; curtain gas (CUR), 40; ionSapary voltage floating (ISVF), +5,500 V. MRM method was used for mass spectrometry quantitative data acquisition.

### Pathogenicity assay.

The WT or mutant sporidia were cultured in 10 mL liquid YePS medium for 2 days, the OD_600_ was adjusted to approximately 1.0, and the sporidia were added to 1 L of fresh YePS without antibiotics at a ratio of 1:100, followed by liquid culture at 28°C, 200 rpm for another 2 days. Such cultured sporidia were mixed in equal volumes with the opposite mating types and used for soaking the sugarcane seed stems, kept at 28°C for 1 day. The soak-inoculated sugarcane stems were planted in pots (5 to 6 seedlings per pot). Two weeks after sugarcane planting, a portion of the sugarcane seedlings were injected with 1 mL of mixed (*MAT-1*×*MAT-2* or *MAT-1*×*ssaro8*Δ) sporidia (OD_600 _≈ 1.0) at the base of the stems. ddH_2_O served as the blank (negative) control for both soaking and injection infections. Each treatment contains around 20 seedlings. Disease symptoms were examined at 3 months post planting. Data analysis was performed by one-way analysis of variance (ANOVA).

### Data availability.

All requisite data from this study are openly available from public repositories and/or online databases as indicated in the manuscript. Additional data may be found in the supplemental material files.
