# Supplementary material for: Aminotransferase SsAro8 Regulates Tryptophan Metabolism Essential for Filamentous Growth of Sugarcane Smut Fungus Sporisorium scitamineum
Source: Microbiol Spectr. 2022 Jul 6;10(4):e00570-22. doi: 10.1128/spectrum.00570-22 (PMC9431617; doi:10.1128/spectrum.00570-22)
Supplement: Supplemental file 1 — Supplemental material. Download spectrum.00570-22-s0001.pdf, PDF file, 1.9 MB [file spectrum.00570-22-s0001.pdf]

Figure S1

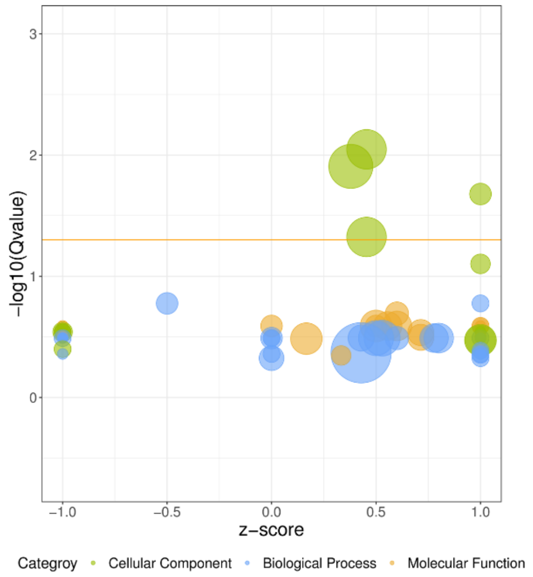

GO term Top 20

ID	Descrption
GO:0006457	protein folding
GO:0006829	zinc II ion transport
GO:0031224	intrinsic component of membrane
GO:0016020	membrane
GO:0005730	nucleolus
GO:0044425	membrane part
GO:0044452	nucleolar part
GO:0048037	cofactor binding
GO:0050662	coenzyme binding
GO:0005215	transporter activity
GO:0015103	inorganic anion transmembrane transporter activity
GO:0022857	transmembrane transporter activity
GO:0046906	tetrapyrrole binding
GO:0022892	substrate−specific transporter activity
GO:0008509	anion transmembrane transporter activity
GO:0009881	photoreceptor activity
GO:0016679	oxidoreductase activity, acting on diphenols and related substances as donors
GO:0016682	oxidoreductase activity, acting on diphenols and related substances as donors, oxygen as acceptor
GO:0044620	ACP phosphopantetheine attachment site binding
GO:0051192	prosthetic group binding

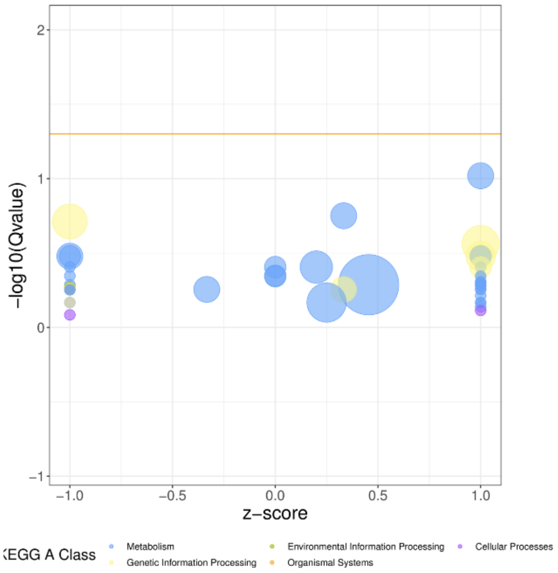

Pathway Top 20

ID	Descrption
ko00910	Nitrogen metabolism
ko00360	Phenylalanine metabolism
ko00130	Ubiquinone and other terpenoid−quinone biosynthesis
ko00290	Valine, leucine and isoleucine biosynthesis
ko00400	Phenylalanine, tyrosine and tryptophan biosynthesis
ko00260	Glycine, serine and threonine metabolism
ko00790	Folate biosynthesis
ko00261	Monobactam biosynthesis
ko00350	Tyrosine metabolism
ko01230	Biosynthesis of amino acids
ko00073	Cutin, suberine and wax biosynthesis
ko01212	Fatty acid metabolism
ko00061	Fatty acid biosynthesis
ko00592	alpha−Linolenic acid metabolism
ko00071	Fatty acid degradation
ko04141	Protein processing in endoplasmic reticulum
ko03010	Ribosome
ko03008	Ribosome biogenesis in eukaryotes
ko03020	RNA polymerase
ko04122	Sulfur relay system

Figure S1 GO and KEGG enrichment of DEGs responsive to PheOH.

Figure S2

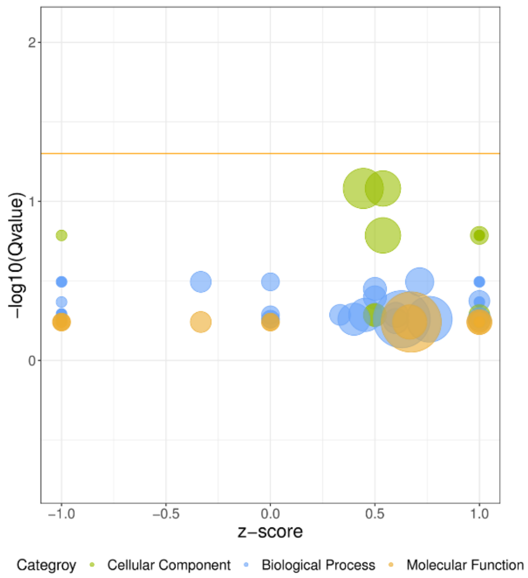

GO term Top 20

ID	Descrption
GO:0006457	protein folding
GO:0006997	nucleus organization
GO:0006359	regulation of transcription from RNA polymerase III promoter
GO:0006998	nuclear envelope organization
GO:0016480	negative regulation of transcription from RNA polymerase III promoter
GO:0019630	quinate metabolic process
GO:0030522	intracellular receptor signaling pathway
GO:0016020	membrane
GO:0031224	intrinsic component of membrane
GO:0044425	membrane part
GO:0005788	endoplasmic reticulum lumen
GO:0005730	nucleolus
GO:0005635	nuclear envelope
GO:0005637	nuclear inner membrane
GO:0017053	transcriptional repressor complex
GO:0031229	intrinsic component of nuclear inner membrane
GO:0031965	nuclear membrane
GO:0044453	nuclear membrane part
GO:0090568	nuclear transcriptional repressor complex
GO:0090571	RNA polymerase II transcription repressor complex

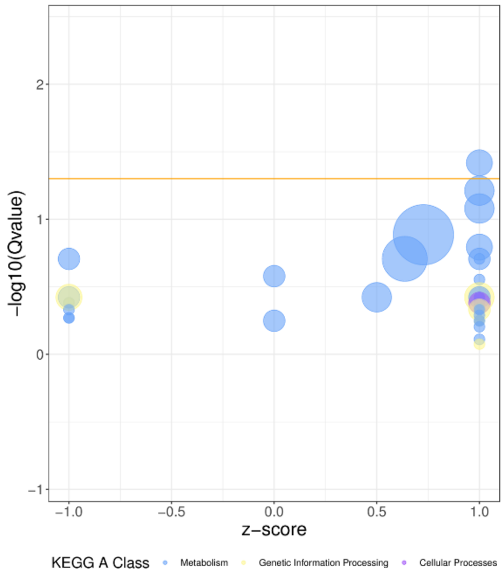

Pathway Top 20

ID	Descrption
ko00910	Nitrogen metabolism
ko00071	Fatty acid degradation
ko00280	Valine, leucine and isoleucine degradation
ko01100	Metabolic pathways
ko01212	Fatty acid metabolism
ko00232	Caffeine metabolism
ko00360	Phenylalanine metabolism
ko00790	Folate biosynthesis
ko01110	Biosynthesis of secondary metabolites
ko00350	Tyrosine metabolism
ko00261	Monobactam biosynthesis
ko01230	Biosynthesis of amino acids
ko00460	Cyanoamino acid metabolism
ko00592	alpha−Linolenic acid metabolism
ko00260	Glycine, serine and threonine metabolism
ko00520	Amino sugar and nucleotide sugar metabolism
ko00300	Lysine biosynthesis
ko04122	Sulfur relay system
ko03010	Ribosome
ko04141	Protein processing in endoplasmic reticulum

Figure S2 GO and KEGG enrichment of DEGs responsive to TyrOH.

Figure S3

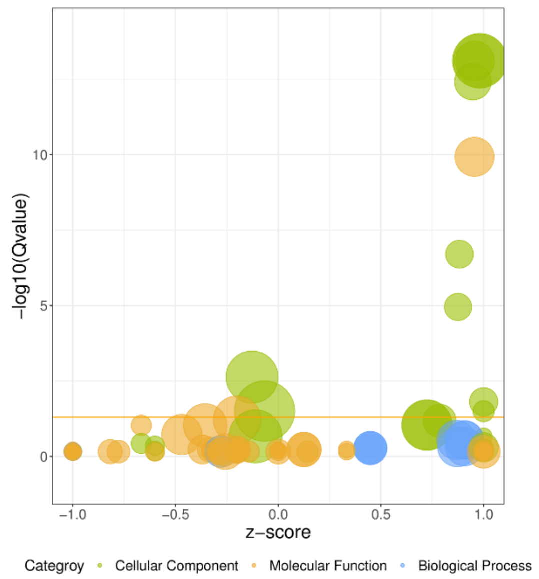

GO term Top 20

ID	Descrption
GO:0005840	ribosome
GO:0030529	intracellular ribonucleoprotein complex
GO:1990904	ribonucleoprotein complex
GO:0044391	ribosomal subunit
GO:0015935	small ribosomal subunit
GO:0015934	large ribosomal subunit
GO:0031224	intrinsic component of membrane
GO:0030684	preribosome
GO:0016020	membrane
GO:0000313	organellar ribosome
GO:0005739	mitochondrion
GO:0043228	non−membrane−bounded organelle
GO:0043232	intracellular non−membrane−bounded organelle
GO:0000314	organellar small ribosomal subunit
GO:0044425	membrane part
GO:0005198	structural molecule activity
GO:0016491	oxidoreductase activity
GO:0016701	oxidoreductase activity, acting on single donors with incorporation of molecular oxygen
GO:0005215	transporter activity
GO:0022857	transmembrane transporter activity

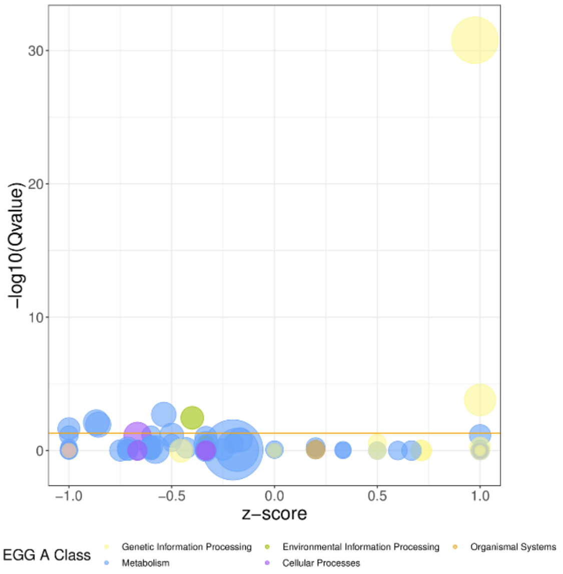

Pathway Top 20

ID	Descrption
ko00410	beta−Alanine metabolism
ko00071	Fatty acid degradation
ko01212	Fatty acid metabolism
ko00360	Phenylalanine metabolism
ko00860	Porphyrin and chlorophyll metabolism
ko00380	Tryptophan metabolism
ko00061	Fatty acid biosynthesis
ko00430	Taurine and hypotaurine metabolism
ko00350	Tyrosine metabolism
ko00620	Pyruvate metabolism
ko00460	Cyanoamino acid metabolism
ko00073	Cutin, suberine and wax biosynthesis
ko00590	Arachidonic acid metabolism
ko00053	Ascorbate and aldarate metabolism
ko00280	Valine, leucine and isoleucine degradation
ko02010	ABC transporters
ko04146	Peroxisome
ko03010	Ribosome
ko03008	Ribosome biogenesis in eukaryotes
ko04122	Sulfur relay system

Figure S3 GO and KEGG enrichment of DEGs responsive to TryOH.

Figure S4

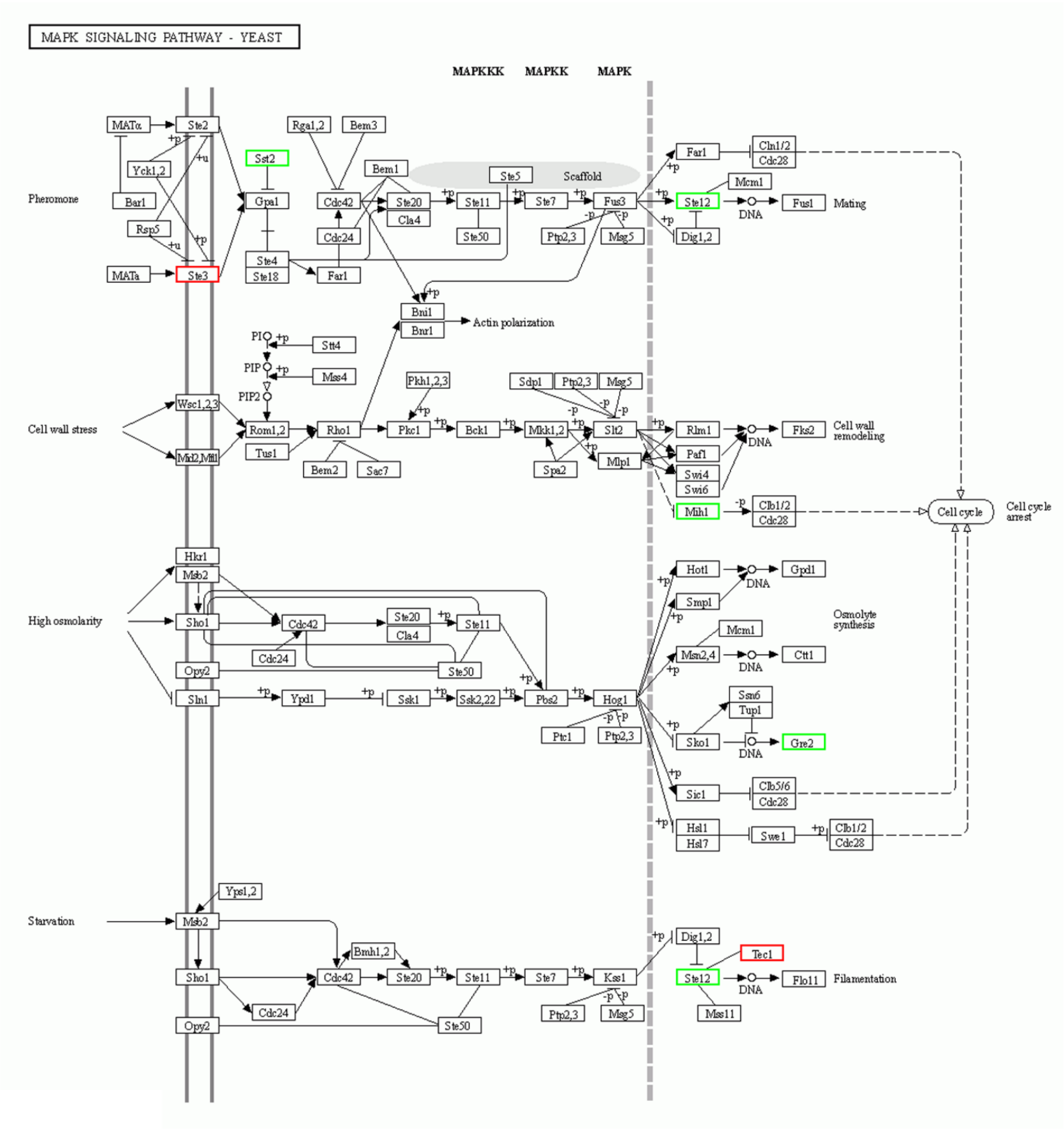

Figure S4 KEGG map of MAPK signaling pathway in response to TryOH treatment. Red and green boxes denote up- and down- regulated genes, respectively, in TryOH-treated vs un-treated sporidia.

Figure S5

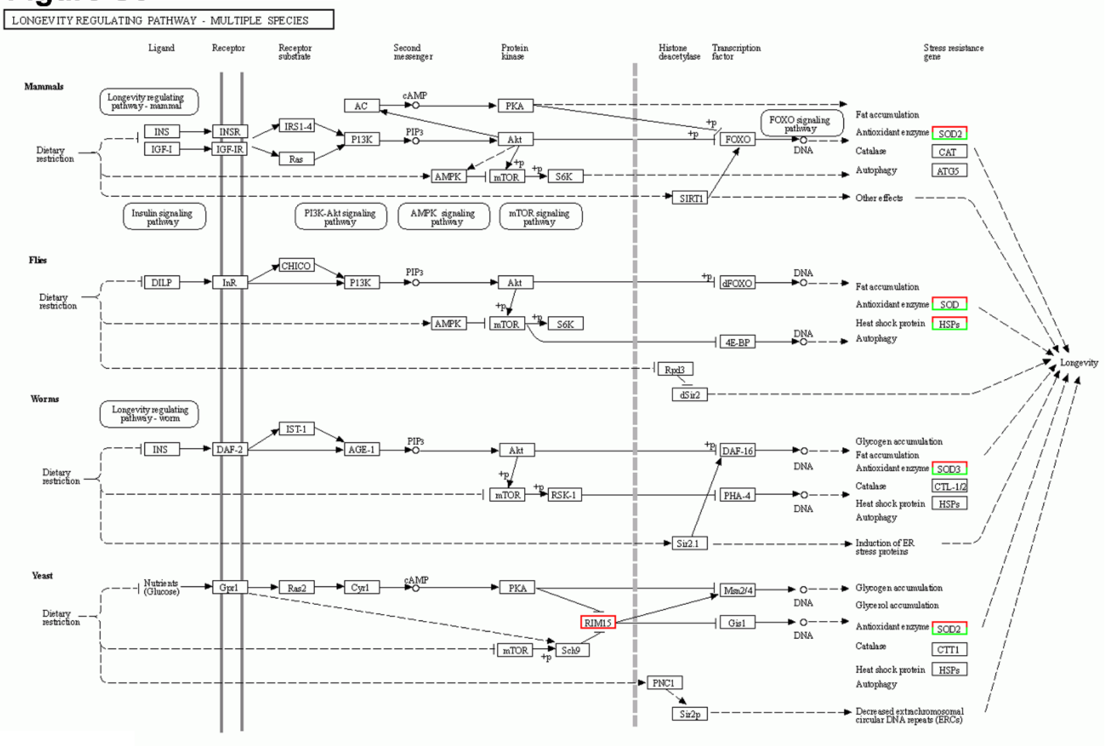

Figure S5 KEGG map of the AGC (Rim15) signaling pathway in response to TryOH treatment. Red and green boxes denote up- and down- regulated genes, respectively, in TryOH-treated vs un-treated sporidia.

Figure S6

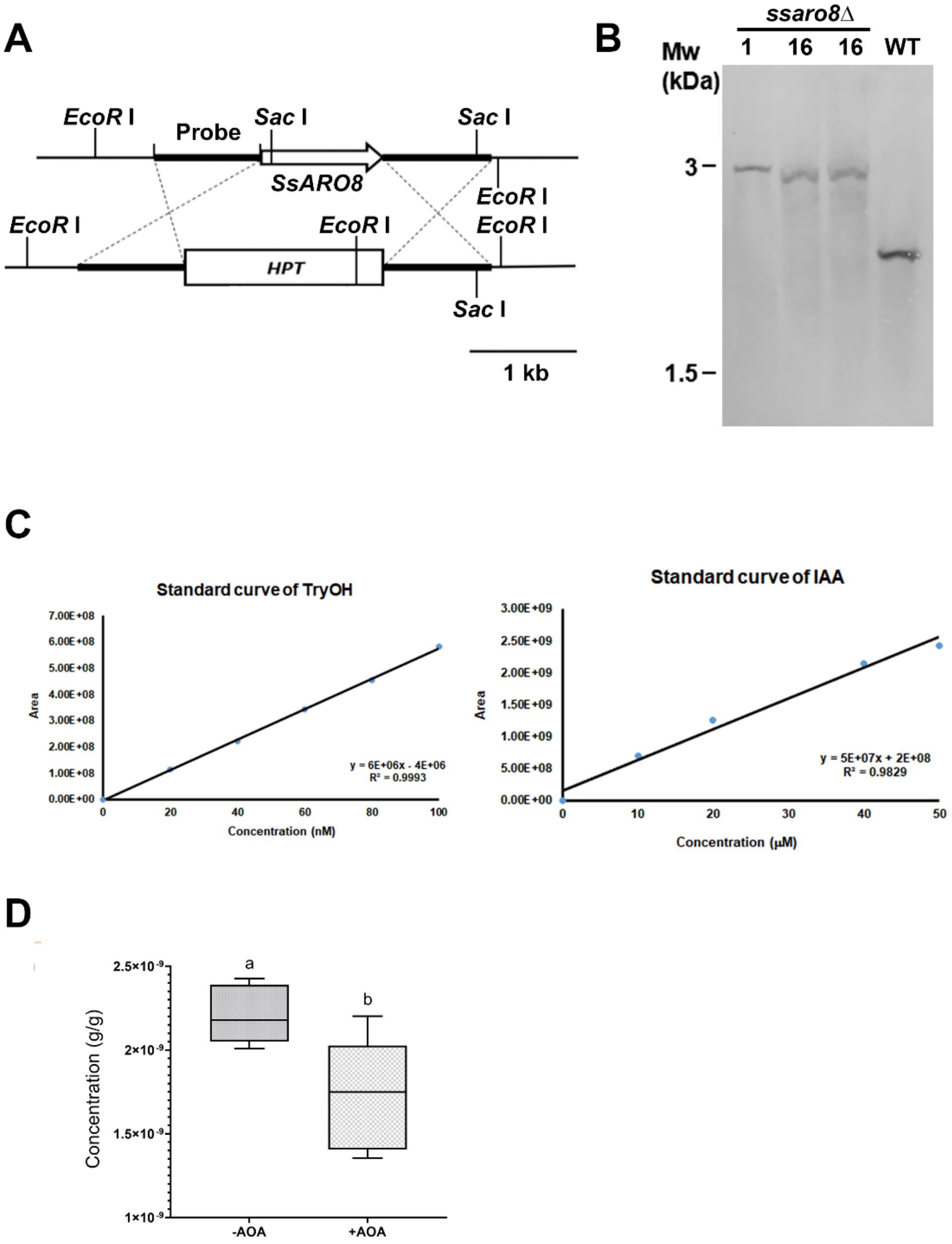

Figure S6 (A) Schematic representation of gene deletion strategy. Dashed lines denote homologous recombination occurring in the franking regions of *SsARO8* coding sequence, resulting in replacement of hygromycin phosphotransferase cassette (*HPT*) in the genome. The restriction enzyme sites and the DNA fragment used as probe for Southern blotting analysis were labeled in the scheme. Scale bar = 1 kb. (B) Southern blotting analysis for verification of *ssaro8*Δ mutants. Detection of a single band of approximately 3 kb, and concomitantly disappearance of the wild-type band (1.6 kb)

was diagnostic as the *ssaro8*Δ mutants. (C) Standard curve of TryOH and IAA using serial diluted TryOH (Sigma, 188255) or IAA (Sigma, I2886) solution. (D) Intracellular level of IAA in the WT sporidia under un-treated (-AOA) or AOA-treated (+AOA) condition was detected by LC-MS, and calculated based on the standard curve as in (C). Different letters denote significant difference (p<0.05). n=8 for each condition, over two biological repeats.

Figure S7

A

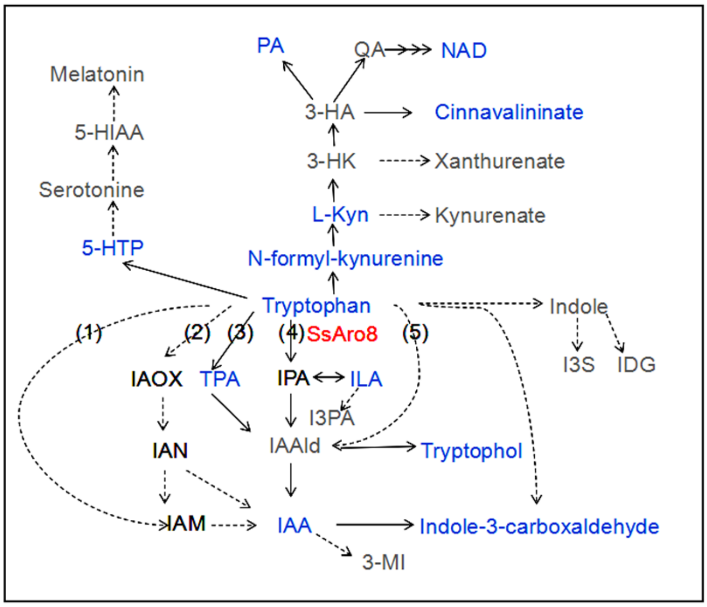

B

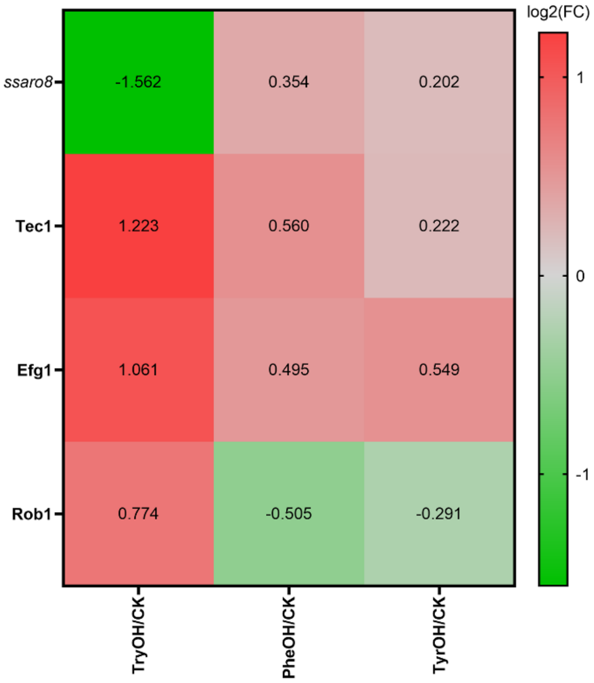

Figure S7 (A) Proposed tryptophan catabolism pathways in *S. scitamineum* based on targeted metabolomics analysis. Trp-dependent IAA biosynthesis pathways include: (1) IAM; (2) IAN; (3) TPA; (4) IPA; (5) TSO pathway. Presence of pathways (solid lines) was inferred based on detection of the intermediate metabolites (blue font), while

undetectable metabolites (grey font) indicate that the corresponding pathways may not be existed (dashed line). IAOX, IAN, IAM, and IPA (black font) were not assessed due to technical limits, so that we could not conclude whether the corresponding biosynthesis pathways exist in *S. scitamineum*. SsAro8 may catalyze Trp to IPA conversion based on comparison between wild-type and *ssaro8*Δ sporidia in terms of TryOH and IAA contents. (B) Heatmap depicting differentiatially expression of selected genes encoding the known biofilm formation regulators under aromatic alcohol treatment conditions. Scale bar represents log2FC values.

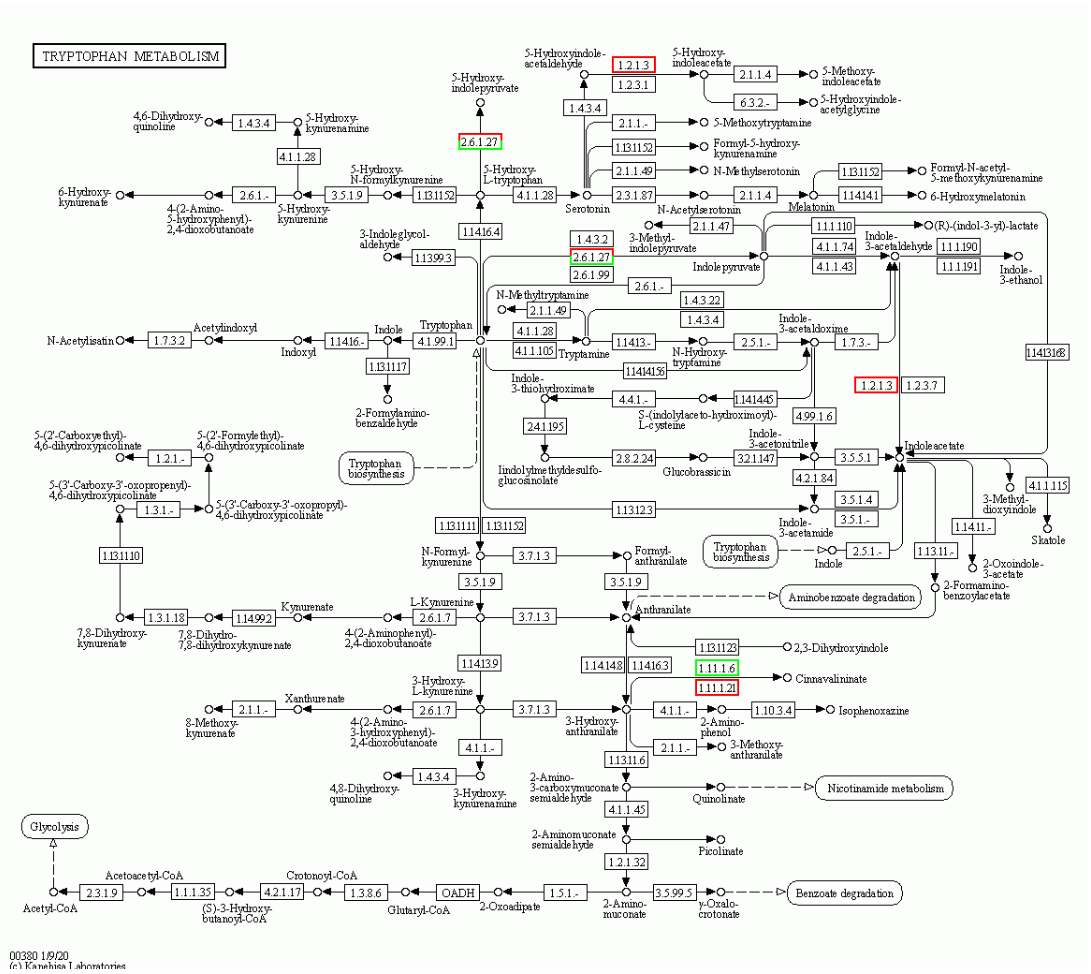

Figure S8 KEGG map of tryptophan metabolism pathway differentially regulated in the *ssaro8*Δ mating culture in comparison to WT mating culture. Red and green boxes denote up- and down- regulated genes, respectively.

Table S1 Primers used in this study.

**Name**	**Sequence (5'→3')**	**Description**
SsARO8-UP-F	5'-AGATGAGGGCAGGCGAAGGTTGGT-3'	
SsARO8-UP-R	5'-TCAGCAAGATCTGCGCAAGTGTCGGTAGCCTTTGGA-3'	
SsARO8P2-UP-F	5'-CGACACTTGCGCAGATCTTGCTGATAGGCAGG-3'	PCR amplification for fused fragment
SsARO8P2-DOWN-R	5'-GCATCAGTTACATAATTCGGGGGATCTGGAT-3'	used for targeted deletion of *SsARO8*
SsARO8-DOWN-F	5'-TCCCCCGAATTATGTAACTGATGCAGCTGGCGAATT-3'	gene
SsARO8-DOWN-R	5'-ATCAAGTCTTTGGTGACGCAGCCTCGC-3'	
HPT-R	5'-GGTCAAGACCAATGCGGAGC-3'	
HPT-F	5'-GCTGCGGCCGATCTTAGCCA-3'	
SsARO8-TG-RBF-F	5'-TTCCGGATCCGTCTCCCACT-3'	PCR verification of *SsARO8* deletion
SsARO8-TG-RBF-R	5'-TCTTAACCAGCCATCACCAC-3'	
SsARO8-PROBE-F	5'-TGTAACTGATGCAGCTGGCGAATT-3'	PCR amplification for probe used in
SsARO8-PROBE-R	5'-ATCAAGTCTTTGGTGACGCAGCCTCGC-3'	Southern blotting analysis
